# Important adverse events to be evaluated in antidepressant trials and meta-analyses in depression: a large international preference study including patients and healthcare professionals

**DOI:** 10.1136/ebmental-2021-300418

**Published:** 2022-07-29

**Authors:** Astrid Chevance, Anneka Tomlinson, Philippe Ravaud, Suzanne Touboul, Catherine Henshall, Viet-Thi Tran, Andrea Cipriani

**Affiliations:** 1 CRESS, INSERM, INRA, F-75004 Paris, France, University of Paris, Paris, France; 2 Department of Psychiatry, University of Oxford, Oxford, UK; 3 Oxford Health NHS Foundation Trust, Warneford Hospital, Oxford, UK; 4 Department of Epidemiology, Columbia University Mailman School of Public Health, New York City, New York, USA; 5 Patient representative, University of Paris, Paris, France; 6 Faculty of Health and Life Sciences, Oxford Brookes University Faculty of Health and Life Sciences, Oxford, UK

**Keywords:** adult psychiatry, depression & mood disorders

## Abstract

**Background:**

Non-serious adverse events (NSAEs) should be captured and reported because they can have a significant negative impact on patients and treatment adherence. However, the reporting of NSAEs in randomised controlled trials (RCTs) is limited.

**Objective:**

To identify the most important NSAEs of antidepressants for patients and clinicians, to be evaluated in RCTs and meta-analyses.

**Methods:**

We conducted online international surveys in English, German and French, including (1) adults prescribed an antidepressant for a depressive episode and (2) healthcare professionals (HCPs) prescribing antidepressants. Participants ranked the 30 most frequent NSAEs reported in the scientific literature. We fitted logit models for sets of ranked items and calculated for each AE the probability to be ranked higher than the least important AE. We also identified additional patient-important AEs not included in the ranking task via open-ended questions.

**Findings:**

We included 1631 patients from 44 different countries (1290 (79.1%) women, mean age 39.4 (SD 13), 289 (37.1%) with severe depression (PHQ-9 score ≥20)) and 281 HCPs (224 (79.7%) psychiatrists). The most important NSAEs for patients were insomnia (95.9%, 95% CI 95.2% to 96.5%), anxiety (95.2%, 95% CI 94.3% to 95.9%) and fatigue (94.6%, 95% CI 93.6% to 95.4%). The most important NSAEs for HCPs were sexual dysfunction (99.2%, 95% CI 98.5% to 99.6%), weight gain (98.9%, 95% CI 97.7% to 99.4%) and erectile problems (98.8%, 95% CI 97.7% to 99.4%). Participants reported 66 additional NSAEs, including emotional numbing (8.6%), trouble with concentration (7.6%) and irritability (6%).

**Conclusions:**

These most important NSAEs should be systematically reported in antidepressant trials.

**Clinical implications:**

The most important NSAEs should contribute to the core outcome set for harms in depression.

**WHAT IS ALREADY KNOWN ON THIS TOPIC**

- Adverse events (AEs) are predictors of lower patient adherence to antidepressant treatment and of poor depression outcomes.
- Despite the large number of clinical trials on antidepressants, there is still a lack of reliable evidence about the safety profile of these medications.

**WHAT THIS STUDY ADDS**

- We conducted online international surveys) to rank the most important AEs according to patients and healthcare professionals (HCPs).
- Overall, 1631 patients from 44 different countries and 281 HCPs were included.
- Patients reported insomnia as the most important, followed by anxiety and fatigue. HCPs ranked sexual dysfunction as the most important AE, followed by weight gain and erectile problems.
- We also identified 66 additional clinically important AEs such as emotional numbing, trouble with concentration, irritability and withdrawal syndrome.

**HOW THIS STUDY MIGHT AFFECT RESEARCH, PRACTICE OR POLICY**

- Non-serious AEs that should be routinely evaluated in trials and meta-analyses about antidepressants in major depression and calls for the development of a core outcome set for harms outcomes in depression.
- These results highlight the importance of engaging in discussions with patients and service users’ caregivers in real-world clinical setting.
- Clinicians should have access to appropriate tools that help engage in collaborative deliberation.

## Background

Depression is experienced by up to 18% of individuals in the general population during their lifetime, with a high morbidity and mortality burden worldwide.^1^ The global incidence of depression has increased from 172 million in 1990 to 258 million in 2017, representing a total increase of 49.9%.^2^ International guidelines recommend antidepressant treatment as the first-line treatment for moderate and severe depression and for persisting mild depression.^3 4^ In the Organisation for Economic Co-operation and Development (OECD) countries, the average use of antidepressants has doubled between 2000 and 2017, going from 31 to 63 defined daily doses/1000 people/day.^5^

Adverse events (AEs) are defined as ‘any untoward medical occurrence in a patient administered a pharmaceutical product and which does not necessarily have to have a causal relationship with this treatment’.^6^ Despite the large number of randomised controlled trials (RCTs) on antidepressants, most meta-analyses have focused on their comparative efficacy, with a minority only focusing on specific AEs.^7^ This lack of information is problematic because AEs are predictors of poorer adherence to antidepressant treatment and poorer clinical outcomes.^8^

Among AEs, there is a distinction between serious and non-serious adverse events (NSAEs). Serious AEs are defined by the Food and Drug Administration as AEs resulting in death, life-threatening, requiring hospitalisation or prolonging an existing hospitalisation, resulting in persistent or significant disability, causing a congenital anomaly/birth defect, requiring specific intervention to prevent permanent impairment or damage.^9^ All other AEs are labelled as non-serious, yet patients and clinicians can still find them troublesome, with consequent implications on the choice of the intervention. NSAEs are not systematically collected and reported in RCTs, which is likely to bias the interpretation of treatment effects, limiting the ability to reliably synthesise information about AEs in meta-analysis.^10 11^

### Objective

This study aimed to rank NSAEs of antidepressants according to people with lived experience of depression and antidepressant treatments, as well as healthcare professionals (HCPs) with direct experience of prescribing antidepressants, to inform the selection of AEs to be investigated in clinical studies and meta-analyses of AEs.^12^

## Methods

We conducted an online survey (available in English, German and French) asking patients and HCPs to rank the NSAEs according to their perceived importance. In this study, NSAEs were considered important if patients considered them as not tolerable from their personal perspective or if HCPs reported them as troublesome for patients according to their clinical experience. Full information about the methods of this study is reported in the published protocol.^13^

### Participants and recruitment

Participants (patients and HCPs) had to speak English, French or German to participate, regardless of their nationality. The surveys recruited participants on a voluntary basis and without payment. All participants provided online informed consent. Both surveys were approved by the Institutional Review Board CERHUPO.5 (Paris, France, IRB 00011928) and registered in the INDS (Institut National des Données de Santé, which is the regulatory body for health data in France) in accordance with the European General Data Protection Regulation.

We recruited adult patients (>18 years old) who were treated with antidepressant medication for unipolar depression.^14^ Patients were excluded if they (1) had a diagnosis of bipolar disorder, (2) reported no current or previous antidepressant exposure or (3) did not use any of the antidepressants listed in the survey. HCPs were recruited if they had experience of prescribing and monitoring antidepressants in patients with depression (eg, psychiatrists, general practitioners/family doctors, hospital doctors, prescribing nurses or prescribing pharmacists). Patients and HCPs were invited to participate through (1) advertisements on social media networks or posted by professional associations and in scientific journals, (2) recruitment campaigns coordinated by the Mental Elf (https://oxfordhealthbrc.nihr.ac.uk/susana-survey/), (3) patient associations or professional networks, and (4) invitations from the ComPaRe e-cohort (https://compare.aphp.fr/) and the MoodNetwork (https://moodnetwork.org/). Full information about the recruitment strategy is reported in online supplemental material 1.

10.1136/ebmental-2021-300418.supp1 Supplementary data

### Development of the survey and survey content

We developed two versions of the international online survey: a patient version and an HCP version. Both versions of the survey had three sections: the collection of descriptive data, the ranking task and an open-ended question to identify further important NSAEs not included in the ranking task.

In the first section of the survey, patients provided information on their sociodemographic and clinical status (eg, age, gender, country, education, severity and duration of symptoms, history of suicidality, current or previous antidepressant treatment, total duration of exposure to antidepressant treatment, and change of treatment due to AEs). HCPs provided demographic, personal and professional information (eg, gender, age, country, profession, experience, including history of depression, experience of antidepressants and AEs, if any).

In the second section, all participants performed the ranking task and were asked to rank the 30 most frequent NSAEs reported in the antidepressant trials using an existing list of drugs from the scientific literature (online supplemental material 2).^14^ First, from the initial list of the 30 most frequent NSAEs, each participant selected the 15 AEs they felt were the most important. Second, the participants sorted these 15 AEs within a constrained template in a tiered system: only one AE could be ranked as the most important (first position), two AEs in the second position, three in the third, four in the fourth and the last five AEs in the fifth position (ie, these are the five least important AEs among the 15 initially selected). This method is validated and aims to shortlist the number of significant items to be ranked by each participant, reducing the burden and increasing the reliability of the evaluation process.^15^

In the third section, all patients and only HCPs who had taken antidepressants were asked to answer an open-ended question to identify additional important AEs not included in the 30 most common NSAEs.

The surveys (originally written in English and then translated into German and French) were codeveloped by clinicians, epidemiologists, social scientists and people with lived experience of depression, and double-checked for clarity and appropriate wording by the Patient and Public Involvement Group from the Oxford Health Biomedical Research Centre and by French-, English- and German-speaking patients and HCPs. Surveys were available on a secured online platform (http://clinicalepidemio.fr/proceed2/en/). The surveys (in English) are reported in online supplemental material 3 and 4 (the French and German translations are available on request from the authors).

### Analysis of survey data

We separately analysed data from patients and HCPs. As per protocol,^13^ data from HCPs who had taken antidepressants for depression were analysed within the HCP group because they participated in the HCP version of the survey.

We used logit models for sets of ranked items to obtain a general ranking of the AEs from the survey data.^16^ The logit models calculate the odds for an individual AE to be ranked above an arbitrary reference, here selected as the AE considered the least important by patients (‘cold symptoms’). As odds are not very intuitive, we then transformed odds into percentages (ie, the probability for each specific AE to be ranked higher than the least important AE, in this case cold symptoms).

We used four alternative methods to rank AEs. First, we calculated the mean rank of each AE. Then, we assessed, for each AE, the proportion of participants having ranked it as the most troublesome (top 1), in the top 3 or in the top 6. We compared the ranking obtained with these four alternative methods (mean rank, top 1, top 3 and top 6) to the ranking obtained with the logit model by calculating, for each AE, the absolute value of the difference in the rank obtained with the logit model and with another method, and finally by averaging these values.

To evaluate how patients’ characteristics could impact their ranking, we fitted models involving an interaction with the characteristics tested: gender (men vs women), severity of disease (PHQ-9 score ≥15 vs <15) and status of treatment (currently under antidepressant versus previously under antidepressant). We adjusted for multiple testing by using a Bonferroni correction.

Recognising that our recruitment methods may have led to a non-representative sample of patients taking antidepressants for depression, we performed a sensitivity analysis to evaluate the impact of weighting our sample so as to obtain a similar distribution of gender, age and education as in the European Health Interview Survey using a method of calibration on margins (online supplemental material 5).^17^ Statistical analyses were performed with R V.3.6.1 (http://www.R-project.org).

Open-text data were analysed using an inductive qualitative content analysis approach.^18^ Three independent clinicians/researchers with professional experience of depression and antidepressants double-coded each participants’ responses; that is, they assigned a code to each word or expression describing any AE’s manifestation and/or impact on the participant’s life. Following this, and with the help of a person with lived experience of depression and antidepressant treatment (ST), three independent clinicians/researchers categorised the codes inductively into a list of AEs, taking into account linguistic considerations and clinical judgements.^19^

### Findings

**Figure 1:**
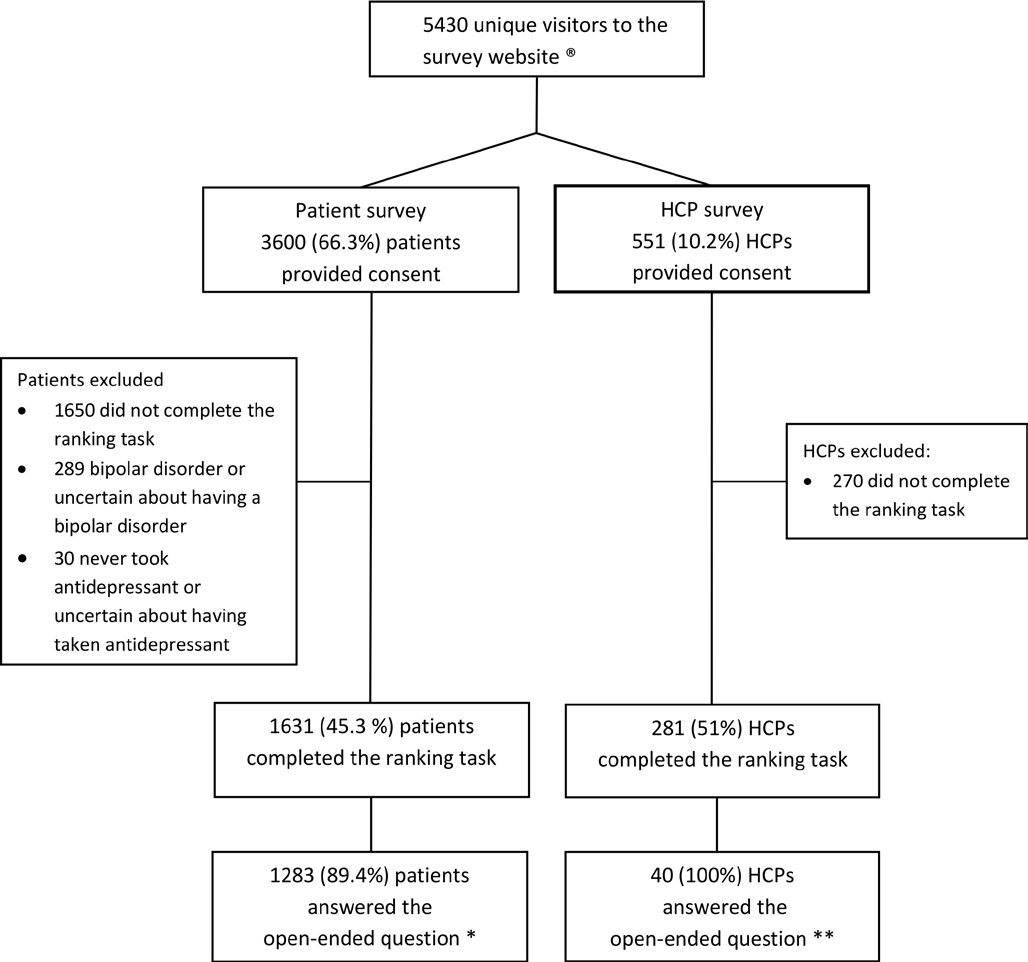
Flowchart for patient and HCP surveys. HCP, healthcare professional. Legend: ® We tracked the number of unique visitors by counting anonymised unique IP address. Each unique visitor may not be eligible (e.g., researchers, curious visitors, same visitor with different IP adress). * The open-ended question was asked to 1435 patients who reported having experienced adverse events of antidepressant. ** The open-ended question was asked to 40 HCPs who reported having themselves experienced adverse events of antidepressant.

From 23 May 2019 to 16 October 2019, 5430 individuals visited the website hosting the online surveys, with 3600 patients and 551 HCPs providing consent to participate. After exclusion of participants who did not complete the ranking task, patients who reported bipolar disorder and/or patients who never took antidepressants, 1631 (45.3%) patients and 281 (51%) HCPs were included in the final analyses (figure 1).

### Characteristics of patients and HCPs

Among the 1631 patients from 44 countries included in the analysis, 797 (48.9%) lived in France and 402 (24.7%) lived in the UK (see table 1 and online supplemental material 6 for full information about individual countries). The mean age of patients was 39.4 (SD 13) years, and 1290 (79.1%) were women. According to the PHQ-9 scores, 269 (16.5%) patients were not depressed (PHQ-9 score <5) when completing the survey, and 605 (37.1%) had a moderate to severe depression (PHQ-9 score ≥15). Regarding the overall number of antidepressants ever taken by each participant, 800 patients (49%) had taken at least three antidepressants and 389 (23.9%) had five or more. The most commonly prescribed antidepressants were fluoxetine (638 patients, 39.1%), venlafaxine (628, 38.5%) and sertraline (602, 36.9%). In terms of AEs, 1438 patients (88.2%) reported having experienced at least one AE; 694 (45.5%) switched antidepressant due to AEs after seeking medical advice, while 248 (16.2%) changed antidepressant without seeking medical advice.

**Table 1:** Characteristics of patients (N=1631)

Patients (N=1631)		
	n (%)	Missing
Gender		0
Female	1290 (79.1)	
Male	312 (19.1)	
Other	29 (1.8)	
Age (years), mean (SD)	39.4 (13.0)	1 (0.1)
Min–max	18–89	
Countries*		16 (1)
France	797 (48.9)	
UK	402 (24.6)	
USA	111 (6.8)	
Canada	107 (6.6)	
Other (40 countries)	198 (12.1)	
Duration of education (years), mean (SD)	17.1 (4.3)	159 (9.7)
Perceived wealth		40 (2.5)
Living comfortably on present income	445 (27.3)	
Coping on present income	605 (37.1)	
Difficult on present income	299 (18.3)	
Very difficult on present income	180 (11.0)	
I prefer not to answer/I don't know	62 (3.8)	
PHQ-9 score		1 (0.1)
Not depressed (<5)	269 (16.5)	
Mild (5–9)	398 (24.4)	
Moderate (10–14)	358 (21.9)	
Moderately severe (15–19)	316 (19.4)	
Severe (≥20)	289 (17.7)	
History of suicide attempt		0
Yes	507 (31.1)	
No	1039 (63.7)	
Prefer not to answer	85 (5.2)	
Antidepressant treatment		
Currently on antidepressant	1139 (69.8)	
Previously on antidepressant	492 (30.2)	
Prescriber of the last antidepressant		
Psychiatrist	903 (55.6)	
Family doctor/general practitioner	681 (41.7)	
Prescribing nurse	11 (0.7)	
Prescribing pharmacist	32 (1.9)	
Other (neurologist, rheumatologist or pain doctor)	4 (0.3)	
Number of antidepressants taken lifelong†		
1	514 (31.5)	
2	317 (19.4)	
3	230 (14.1)	
4	181 (11.1)	
5 or more	389 (23.9)	
Type of antidepressants taken lifelong‡		
Fluoxetine	638 (39.1)	
Venlafaxine	628 (38.5)	
Sertraline	602 (36.9)	
Citalopram	577 (35.4)	
Escitalopram	508 (31.2)	
Paroxetine	402 (24.7)	
Mirtazapine	262 (16.1)	
Amitryptiline	238 (14.6)	
Duloxetine	233 (14.3)	
Exposure to antidepressants		8 (0.5)
Less than 6 months	148 (9.1)	
Between 6 months and 1 year	137 (8.4)	
Between 1 year and 5 years	589 (36.1)	
More than 5 years	745 (45.7)	
I don't know	4 (0.2)	
Experience of AEs		34 (2.1)
Yes	1438 (88.2)	
No	71 (4.4)	
Don’t know	88 (5.4)	
Change of treatment due to AEs		79 (5.2)
Without medical advice	248 (16.2)	
With medical advice	694 (45.5)	
No change	493 (32.3)	
Prefer not to answer	12 (0.8)	

*We report here only the countries representing more than 5% of the patients. The further 38 countries are reported in online supplemental material 6.

†Number of antidepressants reported by patients among the list of 41 antidepressants.

‡We only list antidepressants reported by >10% of the participants. The 31 other antidepressants are reported in online supplemental material 5. Some people may have taken several antidepressants: only participants who reported having experienced AEs (n=1438) or did not know if they experienced AEs (n=88) answered this question (n=1526).

AE, adverse event; PHQ-9, Patient Health Questionnaire-9.

Among the 281 HCPs from 27 countries, 224 (79.7%) were psychiatrists and 35 (12.4%) were general practitioners. Their mean professional experience was 14 (SD 11.4) years (see table 2 and online supplemental material 7 for full information). Among HCPs, 93 (33%) had direct personal experience of depression, and 63 of these (67.7%) were taking or had taken antidepressants. Of these, 40 (63.5%) had experienced AEs.

**Table 2:** Characteristics of HCPs (N=281)

HCPs (N=281)		
	n (%)	Missing
Gender		0
Female	111 (39.5)	
Male	169 (60.1)	
Other	1 (0.4)	
Age (years), mean (SD)	42.4 (12.8)	0
Min–max	22–88	
Profession		0
Psychiatrist	224 (79.7)	
Family doctor/general practitioner	35 (12.4)	
Prescribing pharmacist	3 (1.1)	
Other prescribing physicians	19 (6.8)	
Countries *		2 (0.7)
France	130 (46.3)	
UK	46 (16.4)	
Netherlands	22 (7.8)	
Italy	17 (6.0)	
Other (23)	64 (22.8)	
Professional experience (years), mean (SD)	14.0 (11.4)	1
Min–max	0–45	
Workplace		4 (1.4)
Public hospital	133 (47.3)	
Community health centre	24 (8.6)	
Private practice/private pharmacy	52 (18.5)	
Several workplaces	54 (19.2)	
Other	14 (5.0)	
Have experienced a depressive episode		4 (1.4)
Yes	93 (33.0)	
No	173 (61.4)	
Prefer not to answer	12 (4.3)	
Have ever taken an antidepressant for a depression (n=93)		3 (1.1)
Yes	63 (67.7)	
No	29 (31.2)	
Prefer not to answer	1 (1.1)	
Antidepressants taken lifelong (n=63) †		0
Paroxetine	21 (33.3)	
Citalopram	19 (30.2)	
Escitalopram	16 (25.4)	
Sertraline	14 (22.2)	
Fluoxetine	13 (20.6)	
Has experienced adverse events of antidepressants (n=63)		2 (3.2)
Yes	40 (63.5)	
No	21 (33.3)	

*We only report here the countries representing more than 5% of the HCPs. The further 23 countries are reported in online supplemental material 6.

†We only report here the antidepressant taken by more than 15% of the HCPs. All other antidepressants are presented in online supplemental material 6. Some people may have taken several antidepressants.

HCP, healthcare professional.

### Results of the ranking task of the patients

**Figure 2:**
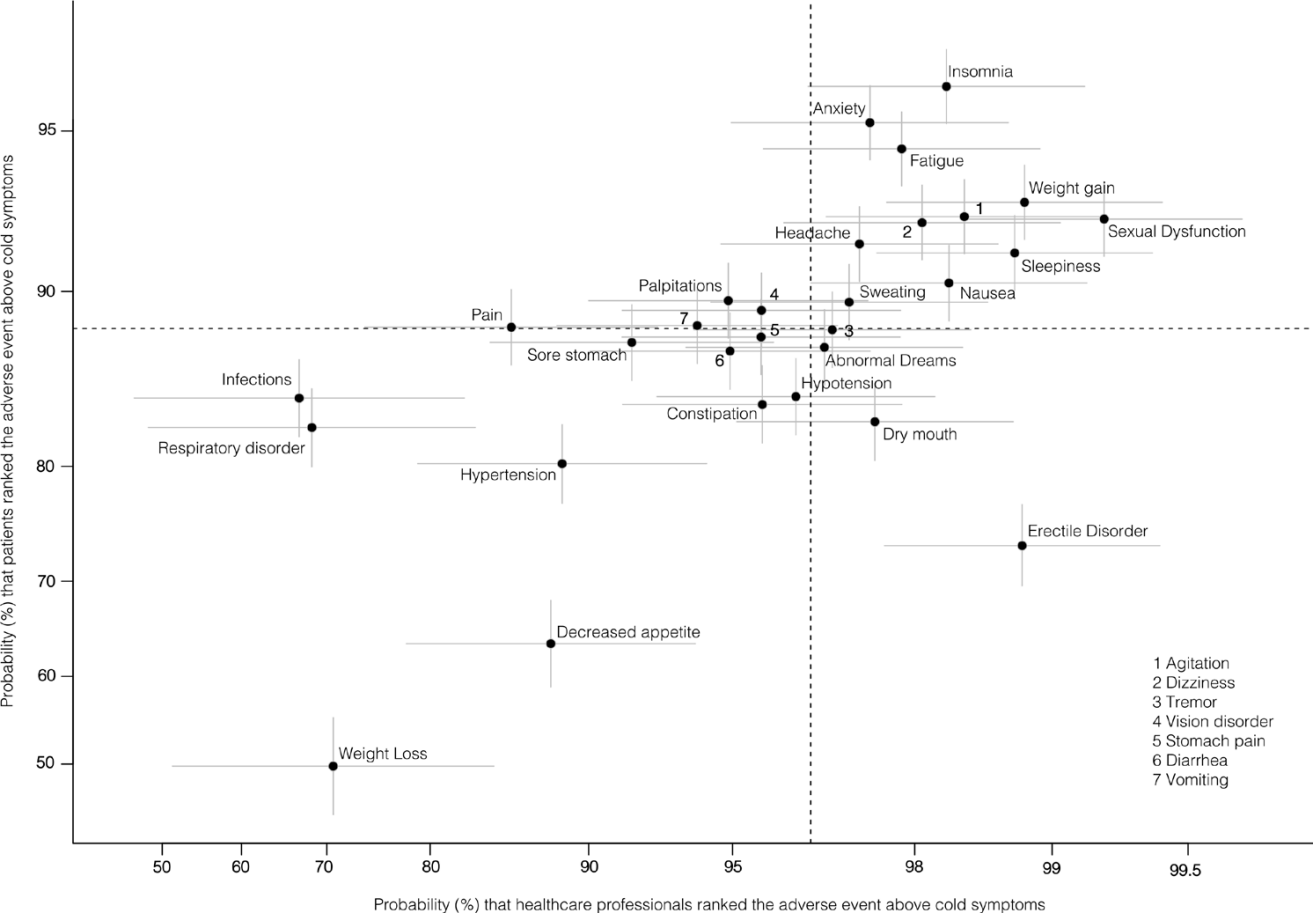
Ranking of AEs of patients and HCPs. Each AE is plotted with the corresponding probability and CI for HCPs (x coordinate) and patients (y coordinate). Cold symptoms are the reference. For instance, the probability of insomnia to be ranked over cold symptoms was 98.3% (95% CI 96.6 to 99.2) for HCPs and 95.9% (95% CI 95.2 to 96.5) for patients. For the sake of visibility, we used a logit scale. The vertical line is the median probability for HCPs (96.6%), and the horizontal line is the median probability for patients (88.3%). The upper right portion contains the AEs considered most important by both patients and HCPs. AE, adverse event; HCP, healthcare professional.

Figure 2 represents each of the 30 NSAEs according to the probability of being ranked above cold symptoms, the least important AE for both patients and HCPs).

The most important AEs for patients were insomnia (95.9%, 95% CI 95.2% to 96.5%), anxiety (95.2%, 95% CI 94.3% to 95.9%), fatigue (94.6%, 95% CI 93.6% to 95.4%), weight gain (93.2%, 95% CI 92.0% to 94.2%), agitation (92.7%, 95% CI 91.5% to 93.8%) and sexual dysfunction (92.6%, 95% CI 91.4% to 93.8%) (table 3 and online supplemental material 8). Use of alternative ranking methods provided similar results for the patients. The average difference of ranking between the logit method and a global method based on (1) the mean rank of each AE; (2) the proportion of patients selecting the AE among the top three most troublesome AEs was 1.3 and 3.7, respectively. We found heterogeneity in the definition of the most troublesome AEs. Especially, no AE was selected among the top three most troublesome AEs by more than 25% of patients in the sample, except insomnia (32.8% of the patients) (online supplemental material 8).

**Table 3:** Most important non-serious AEs of patients and HCPs

AEs	Probability (%) for each specific AE to be ranked higher than the AE considered the least important by patients (ie, cold symptoms) (95% CI)
The six most important AEs for patients	
Insomnia	95.9 (95.2 to 96.5)
Anxiety	95.2 (94.3 to 95.9)
Fatigue	94.6 (93.6 to 95.4)
Weight gain	93.2 (92.0 to 94.2)
Agitation	92.7 (91.5 to 93.8)
Sexual dysfunction	92.6 (91.4 to 93.8)
The six most important AEs for HCPs	
Sexual dysfunction	99.2 (98.5 to 99.6)
Weight gain	98.9 (97.7 to 99.4)
Erectile disorder	98.8 (97.7 to 99.4)
Sleepiness	98.8 (97.6 to 99.4)
Agitation	98.4 (96.9 to 99.2)
Nausea	98.3 (96.6 to 99.2)
**Additional AEs reported in the free-text responses by more than 5% of patients and HCPs who answered the open-ended questions (n=1323**)	**Frequency (%**)
Emotional numbing	8.6
Trouble with concentration	7.6
Irritability	6
Withdrawal syndrome	5.9

AE, adverse event; HCP, healthcare professional.

Gender had a significant impact on the ranking of the following AEs: weight gain, erectile disorder, nausea and sweating (see online supplemental material 9). However, neither severity of depression nor taking or not an antidepressant at the time of the survey had an impact on the ranking (online supplemental material 10 and 11).

The sensitivity analysis using the weighted data set comparable to the European Health Interview Survey on gender, age and education showed no difference in the first three AEs (insomnia, anxiety and fatigue) and small differences with the next four AEs: weight gain (ranked fourth in the raw data set and seventh in the weighted data set), agitation (ranked fifth in both data sets), sexual dysfunction (ranked sixth and fourth, respectively) and dizziness (ranked seventh and fifth, respectively) (online supplemental material 12).^17^

### Comparison of the ranking of patients and HCPs

The most important AEs for HCPs were sexual dysfunction (99.2%, 95% CI 98.5% to 99.6%), weight gain (98.9%, 95% CI 97.7% to 99.4%), erectile disorders (98.8%, 95% CI 97.7% to 99.4%), sleepiness (98.8, 95% CI 97.6 to 99.4), agitation (98.4%, 95% CI 96.9% to 99.2%) and nausea (98.3%, 95% CI 96.6% to 99.2%) (table 3 and online supplemental material 13). Use of alternative ranking methods provided similar results for the HCPs. The average difference of ranks between the logit method and a global ranking based on (1) the mean rank of each AE, and (2) the proportion of patients selecting the AE among the top three most troublesome AEs was 0.4 and 2.4, respectively. We found heterogeneity in the definition of the most troublesome AEs. Only three AEs were cited by more than 25% of patients in the top three most troublesome AEs: sexual dysfunction (38.7%), weight gain (28.1%) and erectile disorder (26.2%) (online supplemental material 13).

The average difference of rank of AEs between the ranking of patients and HCPs (both using the logit model) was 5.4 (online supplemental material 14).

### Additional AEs important for patients and HCPs but not included in the ranking task

In total, 1283 patients and 40 HCPs answered the open-ended question: ‘If you have ever experienced any side effects while taking antidepressant medication, could you please describe these side effects and their impacts on your life, in regard to the potential benefits of the treatment?’ Overall, 66 additional important AEs were identified (online supplemental material 15, Table A). The most frequently cited were emotional numbing (n=154, 11.6%), trouble with concentration (101, 7.6%), irritability (79, 6%) and withdrawal symptoms (78, 5.9%) (table 3). When reporting emotional numbing, participants noted a reduction and/or suppression of all positive and negative feelings. While some patients argue this could be a relief in the first week of the treatment not to feel the intense negative feelings anymore, some complained about the persisting effects, even after treatment cessation.

It had not improved my depression; it had removed my ability to feel sadness (or happiness). This emotional flatness has persisted. (Patient, woman, 39)

This medication made me feel numb for a month and a half. Which was both positive and negative as I felt like I wasn't feeling anything not me. (Patient, woman, 32)

General numbing of feelings, of emotions, which feels vexing in good times but beneficial in bad times. Overall, not being depressed outweighs the problems. (Patient, woman, 36)

Irritability was reported as troublesome, impairing relationships.

I stopped escitalopram because it made me feel angry and lose my temper too much. (Patient, woman, 42)

I had become irritable and angry which affected my family. (Patient, man, 46)

## Discussion

In this study, 1631 patients and 281 HCPs ranked the 30 most common NSAEs reported in RCTs of antidepressants in depression. Among the top 15 most important AEs for patients and HCPs, 11 were common to both groups: insomnia, anxiety, fatigue, weight gain, agitation, sexual dysfunction, dizziness, sleepiness, sweating, headache and nausea. This list was consistent among subgroups of patients, with the exception of ‘erectile problems’, which was ranked as most important by men. A sensitivity analysis using a weighted sample representative of the depressed population in Europe found similar results. Using free-text responses, we also identified additional important AEs not included in the ranking task, such as emotional numbing, trouble with concentration, irritability and withdrawal symptoms.

To our knowledge, this is the first study that ranked the importance of AEs of antidepressants for depression using a large and international sample of patients and prescribing HCPs. Previous studies focused only on the prevalence of AEs, did not evaluate the importance of AEs in patients’ lives, and were limited to individual countries.^20–22^ A review of studies investigated patients’ preferences for medication-associated outcomes in mental disorders and identified no studies dedicated to AEs of antidepressants in depression.^23^

This study has limitations. First, our sample may not be representative of depressed patients taking antidepressants. We found no European data describing the characteristics of people taking antidepressants for depression. There are epidemiological studies about people with depression, but they did not report data about antidepressants’ consumption, or studies about the use of antidepressants, but no information about the diagnosis of patients (eg, depression, anxiety and chronic pain).^24^ Therefore, we decided to use the data from the European Health Interview Survey to weight our sample (on age, gender and education), although it does not entirely represent people taking antidepressants for depression.^17^ This sensitivity analysis found minor differences in the top six AEs ranked by patients. Other characteristics may have affected the representativeness of our sample and thus limit the generalisability of the results of the ranking. For instance, we cannot rule out the possibility that patients who perceive themselves as harmed by antidepressants or who are more vulnerable to AEs may have more likely participated in the survey than patients who do not. However, we were limited by the scarcity of external data describing patients taking antidepressants for depression. There is also a lack of data regarding the determinants of patients’ preferences, such as whether having experienced a given AE has an impact or not on the relative preference for this AE. Second, the surveys were open to any English-speaking, French-speaking or German-speaking patient/HCP without geographical limitations but ultimately involved mostly patients from Western countries. Since the results regard preferences of individuals, they may be sensitive to sociocultural determinants. There is a need to conduct such surveys in other contexts or at least to identify if preferences toward AEs are determined by cultural aspects. Third, the ranking task investigated ‘stated preferences’ of patients, that is, choices including hypothetical scenarios, but they could be different from ‘revealed preferences’, that is, actual choices made by people in real situations. We chose to evaluate stated preference to be closer to the reality of therapeutic decision (before having experienced the treatment/taken the antidepressant), as this is what happens in routine care.^25^ Fourth, we chose to study AEs of antidepressants together as a drug class, even if it is likely that individual antidepressants have different profiles of AEs, both in children, adolescents, adults and older adults.^26^ Other factors can affect the experience of antidepressant treatment, including the dose and duration of the drugs, whether the AEs are during treatment or are residual symptoms, or whether people are taking antidepressants as monotherapy or combination treatment. Fifth, our results must be interpreted in the sole context of antidepressants in depression only. To obtain a ranking of patients’ preferences toward AEs of antidepressants prescribed for other conditions (such as neuropathic pain or urinary incontinence), we would need to extract NSAEs from trials of antidepressants in these conditions first. Finally, the population of the trials in which we identified the list of AEs may be different from the population of patients included in our survey, not only because some of the antidepressants investigated in the older studies may no longer be in use but also because the population of randomised trials is different from the real-world population, due to their strict eligibility criteria.^27^

Discrepancies between patients and HCPs in the ranking of AEs and the important AEs identified with the qualitative content analysis highlight the importance of engaging in discussions with patients and service users’ caregivers. In our survey, 16.2% of patients reported having changed their treatment without medical advice because of AEs, and some patients described the difficulties they have to talk about AEs with HCPs, with the feeling of not being heard, in particular about sexual dysfunction and emotional numbing. Clinicians need training in a real shared decision-making approach.^28^ They should have access to appropriate tools that help engage in collaborative deliberation, and their clinical practice generally needs to be reorganised around the principles of patient engagement.^28^

## Clinical implications

Nowadays, it is recommended to include the perspective of patients in the selection of efficacy outcomes to be included in trials.^29 30^ Several initiatives such as OMERACT (Outcome Measures in Reheumatology - https://omeracthandbook.org/handbook) and COMET^31^ have updated their recommendations to do the same for harms outcomes. To our knowledge, this is the first study to make the proof of concept of a method to select AEs to be included in trials and to provide a ranking of AEs of antidepressants based on their importance from the point of view of patients and HCPs. These findings could set the foundation of a Core Outcome Set for harms^32^ to be measured in all primary and secondary studies involving antidepressants for depression. These important AEs should be systematically evaluated and reported properly in RCTs and their meta-analysis, in order to provide evidence-based information to support treatment decisions in clinical practice.^33^

## Data Availability

Data are available upon reasonable request. Deidentified quantitative data will be shared upon reasonable request to the authors. Additional documents not already provided in the online supplemental material section (eg, informed consent and ethical approval) will be available on request to the authors.
