# Supplementary material for: Important adverse events to be evaluated in antidepressant trials and meta-analyses in depression: a large international preference study including patients and healthcare professionals
Source: Evid Based Ment Health. 2022 Jul 29;25(e1):e41–8. doi: 10.1136/ebmental-2021-300418 (PMC9811084; doi:10.1136/ebmental-2021-300418)
Supplement: Supplementary data [file ebmental-2021-300418supp001.pdf]

Supplementary Material

Supplementary Material 1: Methods for the recruitment of participants 2

Supplementary Material 2: Identification of the 30 non serious AEs to be included in the ranking task of the survey 6

Sources to identify AEs of antidepressants 6

Identification of AEs reported in antidepressants' trials 6

Classification of AEs of antidepressants identified in trials 7

Identification of the 30 AEs to be included in the survey 7

Supplementary Material 3: Patient's questionnaire 9

Supplementary Material 4: Healthcare professional's questionnaire 16

Supplementary material 5: Calibration of the weighted data set 19

Supplementary material 6 Further characteristics of patients 20

Supplementary Material 7: Further characteristics of healthcare professionals 22

Supplementary Material 8: Rankings of adverse events of antidepressants by patients using different analysis' methods 23

Supplementary Material 9: Comparison of the ranking men and women 25

Supplementary Material 10: Comparison of the ranking of patients with mild and severe depression 26

Supplementary Material 11: Comparison of the ranking of patients with current and previous antidepressant medication. 27

Supplementary material 12 : Results of the sensitivity analysis 28

Supplementary material 13: Rankings of adverse events of antidepressants by HCPs using different analysis' methods 29

Supplementary material 14: Comparison of the ranking of patients and HCPs 31

Supplementary Material 15: Adverse events identified with the qualitative content analysis 32

Chevance A, *et al. Evid Based Ment Health* 2022; 25:e41–e48. doi: 10.1136/ebmental-2021-300418

Supplementary Material 1: Methods for the recruitment of participants

1) Recruitment of patient

We recruited patients with:

1) Advertisements on social networks from our personal account:
   a. Groups dedicated to depression in Facebook (36 groups in English, 3 in German and 15 in French)
   b. A Facebook page dedicated to a previous survey conducted by our team: https://www.facebook.com/proceeddepression/
   c. Twitter
   d. LinkedIn

2) Advertisements by foundations on Facebook and twitter
   - Fondation Pour la Recherche Médicale, a non profit foundation in France to fund research teams and projects (25/06/2019)
   - Fondation Falret, a non profit foundation for mental health in France (24/05/2019)
   - Fondation Pierre Deniker, a non profit foundation for mental health in France (24/05/2019)

3) Invitation by e-mail to patients from partner patient associations
   - France depression : a French association of patients with unipolar or bipolar depression
   - Oxford PPI group

4) the ComPaRe e-cohort. ComPaRe is an e-cohort of 32 000 patients with chronic disorders conducted by the Center of Research for Epidemiology and statistics and Paris Hospital, Paris, France. It includes any French person who report at least one chronic condition. Among them. there were 982 patients who reported a depression in July 2019. We proposed them to participate to the survey in July 2019. www.compare.aphp.fr

5) the Moodnetwork e-cohort. Moodnetwork is an e-cohort for mood disorder conducted by the Massachussetts Hospital, Boston, USA. In August 2019 it included 3000 participants: 2000 with bipolar disorder and 1000 with unipolar depression. An e-mail was sent to the 1000 participants with unipolar depression and an advertisement on facebook was posted on 11/09/2019. https://moodnetwork.org/

Example of communication material

Got depression? Got antidepressants?

One in five adults will experience depression in their life. Antidepressants are a common treatment for depression, but we know little about how patients experience antidepressant side-effects. More knowledge will help improve the treatment of depression.

The SUSANA online study (Survey for Understanding Side effects of ANtidepressants in Adults) aims to identify the most troublesome side effects for patients. In this survey, we ask you to choose and rank 15 side effects you find most troublesome, from a list of 30 common side effects. A few additional questions ask about your personal experience with antidepressants

What do you have to do to help?

- Be at least 18 years old,
- Have taken, or are currently taking antidepressants for depression
- Review information about the study and consent to fill the online questionnaire (second page of the survey),
- Answer the 9 minutes-long anonymous online questionnaire
- You can also help spread the word by sharing the link of the survey website on social networks (twitter, facebook, etc)

To do so, please click here to access the survey website: http://clinicalepidemio.fr/proceed2/en/

Dear all,
Here is an international survey about adverse effects on antidepressants driven by scientific teams of Paris and Oxford.
http://clinicalepidemio.fr/proceed2/en/
It asks patients and prescribers to rank the most common adverse effect according to their burden in daily life.
It also asks for their personal experience of the treatment.
Please answer this anonym survey (<10 min) and help disseminate it on social networks. It has been acknowledged by an International Review Board (ethic comittee)
Thank you very much

6) A digital campaign by The Mental Elf

This report summarises the digital activities undertaken by André Tomlin from the Mental Elf to promote the SUSANA survey which launched in May 2019 and ran until October 2019. He used a variety of online methods to disseminate the survey to people and encourage them to complete it:

Blogs

We published two blogs written by the SUSANA survey team to highlight the survey:

a. Antidepressants and weight gain: long-term population impact #SUSANAsurvey, 24th May 2019. https://www.nationalelfservice.net/treatment/antidepressants/antidepressant-side-effects-susanasurvey/
b. Are antidepressants safe? A new umbrella review of observational studies suggests they are, but we need more accurate data, 4th Oct 2019. https://www.nationalelfservice.net/treatment/antidepressants/are-antidepressants-safe-a-new-umbrella-review-of-observational-studies-suggests-they-are-but-we-need-more-accurate-data/

We also published four other blogs on antidepressants during the time period of the survey. which were also used to promote the SUSANA survey on social media.

Social media

Twitter

We posted a series of tweets encouraging people to complete the SUSANA survey, which began on 17th May 2019 and continued until the survey closed in October 2019. The tweets came from the Mental Elf Twitter account which had >70k followers in May 2019, and increased its following during the duration of the survey.

We asked supporters and followers of the Mental Elf Twitter account to share our tweets from their own accounts, to reach as wide and diverse an audience as possible.

We mentioned specific supporters in tweets to encourage them to complete the survey, and share it with their followers.

Facebook

We share the survey on the Mental Elf Facebook page which had >10k followers.

We shared the survey on relevant Facebook groups and pages to reach the target audience (people who have taken or prescribed antidepressants). We also asked relevant professional groups to post the survey to closed Facebook groups and this happened in a wide number of cases.

LinkedIn

We shared the survey via relevant groups (e.g. psychology, psychiatry, mental health nursing).

Audio/video

We used graphics, audio podcasts and videos to make our tweets more noticeable and shareable.

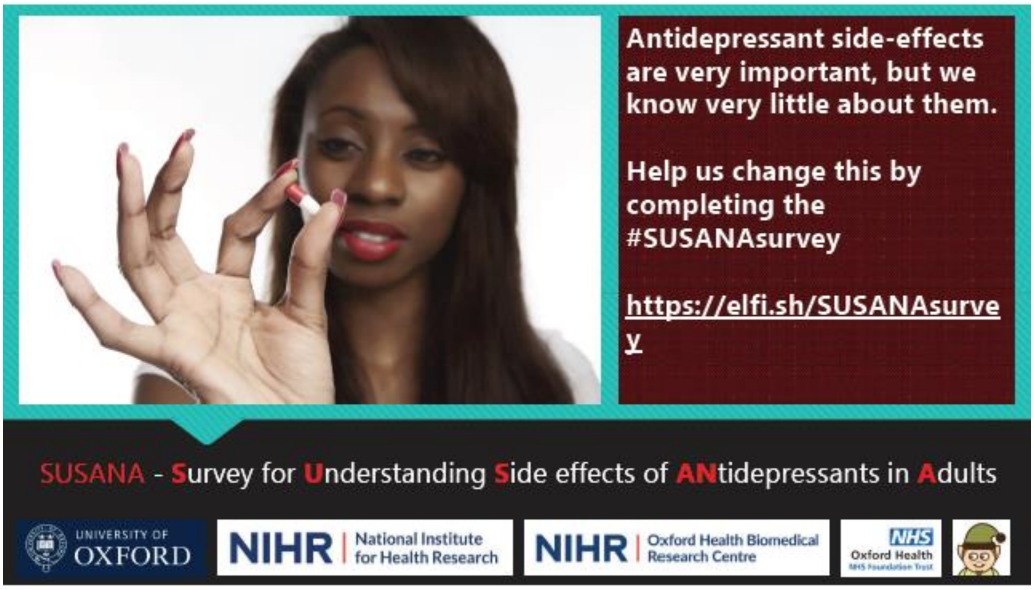

2) Recruitment of healthcare professionals (HCPs)

We recruited HCPs with :

1) Advertisements in specific social networks

   - Professional groups of mental health HCPs on Facebook
   - Twitter
   - LinkedIn
   - ResearchGate

2) Newsletter on websites of professional associations, also send by e-mail

- L'Encéphale (a french society of mental health HCPs)
- Jeunes médecins

3) Editorial in a scientific review:

Kernot C, Tomlinson A, Chevance A, Cipriani A, One step closer to personalised prescribing of antidepressants: using real-world data together with patients and clinicians' preferences, *Evid Based Ment Health* 2019; **22**: 91–2.

Supplementary Material 2: Identification of the 30 non serious AEs to be included in the ranking task of the survey

Sources to identify AEs of antidepressants

We reanalyzed the 522 trials included in a meta-analysis of double-blind randomized controlled trials of 21 active antidepressant drugs provided as monotherapy in the acute phase treatment of major depression[1]. The 21 antidepressants were all second-generation antidepressants approved by the regulatory agencies in the USA, Europe or Japan. They included selective serotonin reuptake inhibitor (SSRIs), selective serotonin and norepinephrine reuptake inhibitor (SNRIs), norepinephrine and dopamine reuptake inhibitor (NDRIs) (agomelatine, bupropion, citalopram, desvenlafaxine, duloxetine, escitalopram, fluoxetine, fluvoxamine, levomilnacipran, milnacipran, mirtazapine, paroxetine, reboxetine, sertraline, venlafaxine, vilazodone, and vortioxetine), tricyclics (amitriptyline and clomipramine), and trazodone and nefazodone, Participants of trials included in the meta-analysis were adults (>18 years) of both sexes with a primary diagnosis of unipolar major depression. All inclusion criteria, methods of selection and screening of these trials are reported in the corresponding paper [1].

This systematic review of antidepressants in depression is currently updated [2].

Identification of AEs reported in antidepressants' trials

Two researchers independently extracted each adverse event reported in included studies (those from Cipriani et al 2018. plus those from the updated search[1.2]. The adverse events and the number of participants experiencing each of them were recorded exactly as reported in the studies. If an adverse was not reported, we considered that no patients experienced it. Any individual discrepancies discussed and resolved between the two researchers, or, if needed, via a consensus decision with a third researcher.

In a second step, we merged clinically similar individual adverse events. Firstly, using the Medical Dictionary for Regulatory Activities (MedDRA) the adverse events were assigned the preferred term (PT), higher level term (HLT) and higher-level group term (HLGT). Secondly, adverse events with same the PT (and HLT and HLGT) were combined and renamed according to the PT. Thirdly, adverse

Chevance A, *et al. Evid Based Ment Health* 2022; 25:e41–e48. doi: 10.1136/ebmental-2021-300418

events were further combined if deemed to be clinically relevant by a team of expert clinicians. Further details on data extraction and classification are detailed in the protocol [2].

We extracted >700 AEs exactly as reported (e.g.. abdominal pain, abdominal pain upper, abdominal discomfort) from the clinical trials. Using the MeDdra classification, we identified >100 different "unique" AEs.

Classification of AEs of antidepressants identified in trials

AEs were classified into serious and non-serious AEs using the classification employed by the US Food and Drug Administration[3]. Serious AEs were those that resulted in death, were life threatening, required hospitalization or prolonged an existing hospitalization, resulted in persistent or significant disability/incapacity, may have caused a congenital anomaly/birth defect, required specific intervention to prevent permanent impairment or damage. All AEs that were not classified as serious AEs were considered as non-serious AEs.

Identification of the 30 AEs to be included in the survey

We summed the number of participants experiencing each non-serious AEs over all trials and included the 30 firsts in the surveys.

These AEs were nausea, headache, dry mouth, insomnia, dizziness, sedation/somnolence, diarrhea, constipation, sexual dysfunction, fatigue, rhinitis/nasopharyngitis, hyperhidrosis, respiratory disorder, anxiety, decreased appetite, tremor, pain, vomiting, abdominal pain/discomfort, dyspepsia, agitation, visual impairment, ejaculation disorder/erectile dysfunction, weight increased, weight decreased, blood pressure increased, arrhythmia/heart rate disorder, abnormal dreams, infection, blood pressure decreased,

References

1 Cipriani A, Furukawa TA, Salanti G. *et al.* Comparative efficacy and acceptability of 21 antidepressant drugs for the acute treatment of adults with major depressive disorder: a systematic review and network meta-analysis. *Lancet Lond Engl* 2018; **391**: 1357–66.

2 Tomlinson A. Efthimiou O. Boaden K. *et al.* Side effect profile and comparative tolerability of 21 antidepressants in the acute treatment of major depression in adults: protocol for a network meta-analysis. *Evid Based Ment Health* 2019; **22**: 61–6.

3 Commissioner O of the. What is a Serious Adverse Event? *FDA* 2019; published online Jan 8. https://www.fda.gov/safety/reporting-serious-problems-fda/what-serious-adverse-event (accessed Aug 26. 2020).

Supplementary Material 3: Patient's questionnaire

All questionnaires are available at this address: http://clinicalepidemio.fr/proceed2/en/indexCache.php

Study title: Antidepressants: Which side effects are the least tolerated?

Researchers
Dr. Astrid CHEVANCE, Prof. Philippe RAVAUD
Dr. Anneka Tomlinson, Prof. Andrea Cipriani
Centre d'Epidémiologie et Statistique Sorbonne Paris Cité (INSERM U1153), Paris, France
Department of Psychiatry, University of Oxford, UK
NIHR Oxford Health Biomedical Research Centre, Warneford Hospital, Oxford, UK

Dear participant,

This scientific study aims to identify what side effects are the least tolerated by patients, It is run by Public Health researchers of the Center of Epidemiology and Statistics Sorbonne Paris Cité (INSERM U1153, Paris, France).

Ready to start the questionnaire?

- ○ **I accept to participate**
- ○ **I do not accept to participate.**

The following questions help to describe your experience of depression.
*Questions marked * require a response in order to proceed to the next page.*

You are:*

- ○ A woman
- ○ A man
- ○ Other gender

Your age* (in years)

Your country of residence *

You consider yourself as* :

- ○ Currently depressed
- ○ I was previously depressed. but I am currently not depressed
- ○ I don't know

How long has this episode of depression lasted (approximately)?*
(in years or months)

Have you ever been diagnosed with bipolar disorder?*

- ○ Yes
- ○ No
- ○ I don't know

Chevance A, *et al. Evid Based Ment Health* 2022; 25:e41–e48. doi: 10.1136/ebmental-2021-300418

Have you ever attempted suicide whilst depressed?*

- Yes
- No
- I prefer not to answer

PHQ-9 questionnaire incorporated :
The following is a scale to help identify possible depressive symptoms.
Over the last 2 weeks, how often have you been bothered by any of the following problems ?

Little interest or pleasure in doing things*

- Never
- Several days
- More than half the days
- Nearly every day

Feeling down, depressed or hopeless*

- Never
- Several days
- More than half the days
- Nearly every day

Trouble failing or staying asleep or, for some, sleeping too much*

- Never
- Several days
- More than half the days
- Nearly every day

Feeling tired or having little energy*

- Never
- Several days
- More than half the days
- Nearly every day

Poor appetite, or, for some, overeating*

- Never
- Several days
- More than half the days
- Nearly every day

Feeling bad about yourself - or that you are a failure or have let yourself or your family down.*

- Never
- Several days
- More than half the days
- Nearly every day

Trouble concentrating on things, such as reading the newspaper or watching television*

- Never
- Several days
- More than half the days
- Nearly every day

Moving or speaking so slowly that other people could have noticed? Or the opposite-being so fidgety or restless that you have been moving around a lot more than usual*

- Never
- Several days
- More than half the days
- Nearly every day

Thoughts that you would be better off dead or of hurting yourself in some way*

- Never
- Several days
- More than half the days
- Nearly every day

If you checked off any problem, how difficult have these problems made it for you to do your work, take care of things at home, or get along with other people?

- Not difficult at all
- Somewhat difficult
- Very difficult
- Extremely difficult

Here is a list of side effects that have been reported by patients taking antidepressants, You may have experienced NONE, SOME or ALL of these side effects.
Please click on the 15 side effects (from the list below), that you think are the most important and that you would least like to experience. (the order of adverse events appeared randomly)

Anxiety
Stomach pain or discomfort
Constipation
Decreased appetite
Sexual dysfunction (decreased sexual desire, disturbance in sexual arousal, orgasm abnormality)
Vomiting
Sweating
Abnormal dreams (in particular nightmare, strange dreams, etc.)
Pain (any kind of body pain excluding headache and stomach pain)
Weight loss
Problems with vision (blurred vision)
Tremor (like hands shaking)
Dry mouth
Insomnia (difficulty falling asleep, early waking, interrupted sleep, reduced sleep, etc.)
Headache (all kinds of headache)
Fatigue (tiredness)

Palpitations, heart beating fast
Erectile problems and/or ejaculation problems
Sleepiness (sedation)
Weight gain
Nausea
Blood pressure increased (hypertension)
Cold symptoms
Respiratory disorder (flu and flu symptoms, cough, breathing difficulties)
Diarrhea
Infections (urine infection, viral infection)
Acid indigestion, acid reflux, sore stomach, heartburn
Dizziness (light-headed, faintness)
Agitation (nervousness, irritability, restlessness)
Faintness in standing, low blood pressure (hypotension and orthostatic hypotension)

Here are the 15 side effects that you have chosen.
Please score them from the least tolerable for patients to the most tolerable (the most acceptable side effects).
Please, give the score 1 to the least tolerable and the score 5 to the most acceptable, Be careful, you can give one "1", two "2" ,three "3", four "4" and five "5". At the bottom pf the page, a validation button will appear as soon as you will have scored all side effects.
Score 1 = the least tolerable side effect ; score 5 = the most tolerable side effects

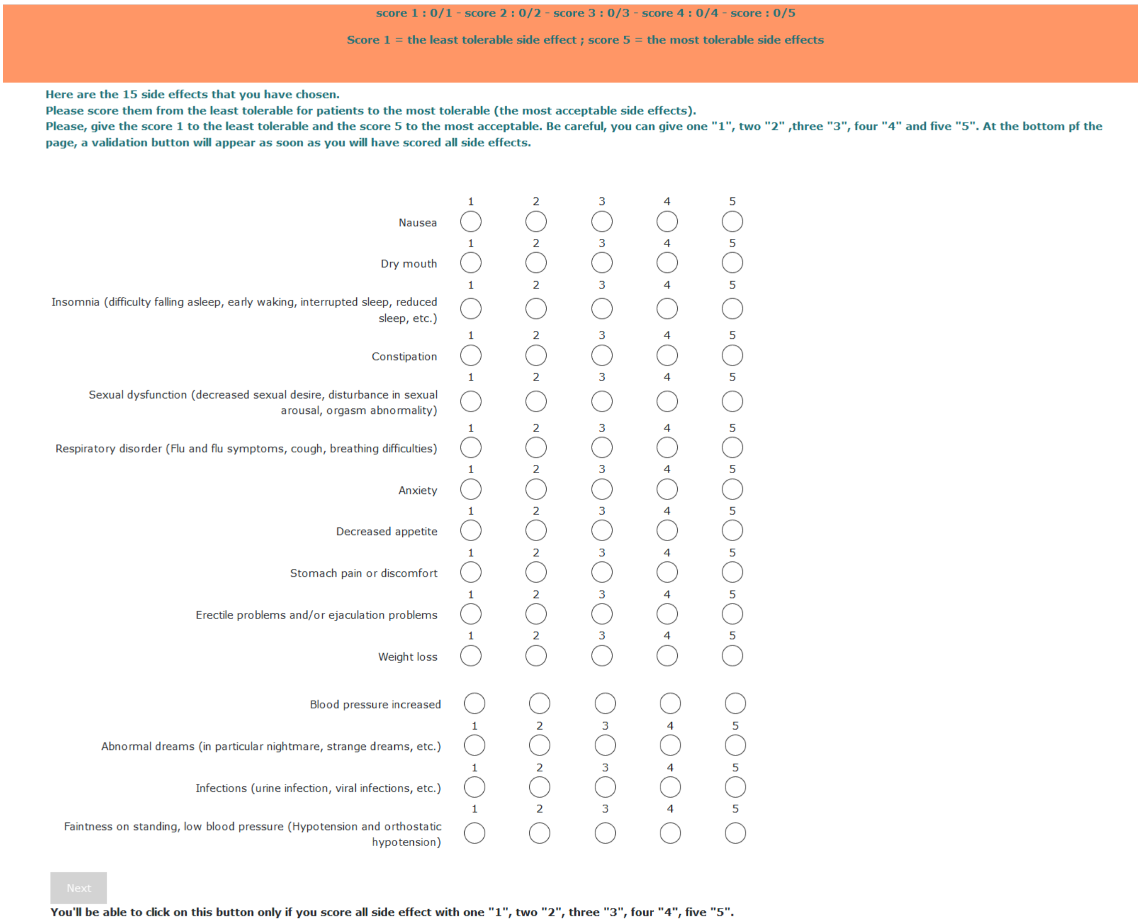
score 1 : 0/1 - score 2 : 0/2 - score 3 : 0/3 - score 4 : 0/4 - score : 0/5

Score 1 = the least tolerable side effect ; score 5 = the most tolerable side effects

Here are the 15 side effects that you have chosen.
Please score them from the least tolerable for patients to the most tolerable (the most acceptable side effects).
Please, give the score 1 to the least tolerable and the score 5 to the most acceptable. Be careful, you can give one "1", two "2" ,three "3", four "4" and five "5". At the bottom pf the page, a validation button will appear as soon as you will have scored all side effects.

	1	2	3	4	5
Nausea	○	○	○	○	○
Dry mouth	○	○	○	○	○
Insomnia (difficulty falling asleep, early waking, interrupted sleep, reduced sleep, etc.)	○	○	○	○	○
Constipation	○	○	○	○	○
Sexual dysfunction (decreased sexual desire, disturbance in sexual arousal, orgasm abnormality)	○	○	○	○	○
Respiratory disorder (Flu and flu symptoms, cough, breathing difficulties)	○	○	○	○	○
Anxiety	○	○	○	○	○
Decreased appetite	○	○	○	○	○
Stomach pain or discomfort	○	○	○	○	○
Erectile problems and/or ejaculation problems	○	○	○	○	○
Weight loss	○	○	○	○	○
Blood pressure increased	○	○	○	○	○
Abnormal dreams (in particular nightmare, strange dreams, etc.)	○	○	○	○	○
Infections (urine infection, viral infections, etc.)	○	○	○	○	○
Faintness on standing, low blood pressure (Hypotension and orthostatic hypotension)	○	○	○	○	○

Next

You'll be able to click on this button only if you score all side effect with one "1", two "2", three "3", four "4", five "5".

Have you ever taken antidepressants to treat depression?*

- Yes I am currently taking antidepressants
- Yes I have taken antidepressant previously, but not at this moment
- No I have never taken antidepressants
- I don't know

Who prescribed the antidepressant for you? (in case of several prescriptions, answer for the most recent) *

- A Psychiatrist
- A General Practitioner
- A prescribing nurse
- A prescribing pharmacist
- Another healthcare professional Please specify :

Which antidepressants have you taken? This may be difficult to remember, so here is a list of antidepressants to help you remember the names of those that you took.
Please select ALL of the antidepressants that you have ever taken in your life.*
This may be difficult to remember, even if you don't remember every treatment, please try to answer as specificly as possible

- Agomelatine (Valdoxan®, Melitor®, Thymanax®)
- Amitriptyline (Elavil®, Laroxyl®, Tryptanol®, Endep®, Elatrol®, Tryptizol®, Trepiline®, Redomex®)
- Amoxapine (Defanyl®)
- Citalopram (Seropram®, Celapram®, Celexa®, Ciazil®, Cilift®, Cipram®, Cipramil®, Ciprapine®, Citabax®, Citalec®, Citaxin®, Citrol®, Ecosol®, Mepha®, Recital®, Talam®, Zentius®, Zetalo®)
- Clomipramine (Anafranil®)
- Desipramine (Pertofran®)
- Deroxat (Paroxetine®)
- Dosulépine (Prothiaden ®)
- Doxépine (Quitaxon®)
- Duloxetine (Cymbalta®, Yentreve®, Xeristar®, AriClaim®)
- Escitalopram (Seroplex®)
- Fluoxetine (Prozac®)
- Fluvoxamine (Floxyfral®)
- Imipramine (Tofranil®)
- Iproniazide (Marsilid®)
- Maprotiline (Ludiomil®)
- Mianserine (Athymil®)
- Minalcipran (Ixel®)
- Mirtazapine (Norset®)
- Moclamine (Moclobemide®)
- Nortiptyline (Sensoval®, Aventyl®, Pamelor®, Norpress®, Allegron®, Noritren®, Nortrilen®)
- Paroxetine (Deroxat®, Paxil®, Seroxat®)

Chevance A, *et al. Evid Based Ment Health* 2022; 25:e41–e48. doi: 10.1136/ebmental-2021-300418

- Phénelzine (Nardelzine®, Nardil®)
- Sertraline (Zoloft®)
- Tianeptine (Stablon®, Coaxi®l, Tatinol®)
- Timipramine (Surmontil®)
- Tranylcypromine (Parnate®)
- Trazodone (Tritticon®, Desyrekn®, Thombarn®, Trialodine®, Trazolan®)
- Venlafaxine (Effexor®)
- Viloxazine (Vivalan®)
- Vortioxetine (Brintellix ®)
- I have never taken any of these drugs
- I think I have taken another antidepressants, please specify :

What is the total time that you have taken antidepressants throughout your life? *
This may be difficult to remember, even if your answer is not the exact time it is very helpful to have a rough estimate

- Less than 6 months
- Between 6 months and a year
- Between 1 and 5 year(s)
- More than 5 years
- I don't know

The following questions focus on your experience of the side effects of antidepressants: Whilst taking antidepressant medication have you ever experienced any side effects?*

- No
- Yes
- I don't know

Could you please describe these side effects and their impacts on your life, in regards to the potential benefits of the treatment?

Writing full sentences is not mandatory, please feel free to write whatever words and ideas you wish.

Did these side effect(s) lead you to change your treatment routine? *

- Yes, I decided with medical advice (e.g. with my doctor) to change my treatment routine (stop, change in dose or switch to a different antidepressant)
- Yes, I decided without medical advice to change my treatment routine (stop or change the dose)
- No, I did not change my treatment routine and took the medication as it was prescribed
- I prefer not to answer

These two final questions will help further describe this study's participants. Please try to answer it as specifically at possible.

Chevance A, *et al. Evid Based Ment Health* 2022; 25:e41–e48. doi: 10.1136/ebmental-2021-300418

At what age did you finish your education? *

If ongoing. please give your current age

Which of these descriptions come closest to how you feel about your household income now?*

- Living comfortably on present income
- Coping on present income
- Difficult on present income
- Very difficult on present income
- I prefer not to answer
- I don't know

Supplementary Material 4: Healthcare professional's questionnaire

Study title: Antidepressants: Which side effects are the least tolerated?

Researchers
Dr. Astrid CHEVANCE, Prof. Philippe RAVAUD
Dr. Anneka Tomlinson, Prof. Andrea Cipriani
Centre d'Epidémiologie et Statistique Sorbonne Paris Cité (INSERM U1153), Paris, France
Department of Psychiatry, University of Oxford, UK
NIHR Oxford Health Biomedical Research Centre, Warneford Hospital, Oxford, UK

Dear participant,

This scientific study aims to identify what side effects are the least tolerated by patients, It is run by Public Health researchers of the Center of Epidemiology and Statistics Sorbonne Paris Cité (INSERM U1153, Paris, France),

Ready to start the questionnaire?

- ○ **I accept to participate**
- ○ **I do not accept to participate**

What is your profession?*

- ○ Psychiatrist/psychiatry trainee
- ○ General Practitioner/GP trainee
- ○ Prescribing nurse
- ○ General Prescribing pharmacist
- ○ Other Please specify : [ ]

You are*

- ○ A woman
- ○ A man
- ○ Other gender

Your age*

Which country do you work in ? *
How many years experience do you have prescribing antidepressants to patients with depression? *
Your current place of work is a: *

- ☐ Public Hospital
- ☐ Community health center
- ☐ Private Practice/Private Pharmacy
- ☐ Other Please specify : [ ]

Here is a list of 30 side effects.
Based on your clinical experience, please click on the 15 side effects that you think are the most clinically relevant (troublesome) for patients.
(the order of Adverse events appeared randomly)

Chevance A, *et al. Evid Based Ment Health* 2022; 25:e41–e48. doi: 10.1136/ebmental-2021-300418

Constipation
Stomach pain or discomfort
Fatigue (tiredness)
Sweating
Abnormal dreams (in particular nightmares, strange dreams, etc,)
Weight loss
Dry mouth
Sexual dysfunction (decreased sexual desire, disturbance in sexual arousal, orgasm abnormality)
Palpitations, heart beating fast
Diarrhoea
Pain (any kind of body pain excluding headache and stomach pain)
Anxiety
Faintness on standing, low blood pressure (Hypotension and orthostatic hypotension)
Problems with vision (blurred vision)
Insomnia (difficulty falling asleep, early waking, interrupted sleep, reduced sleep, etc.)
Agitation (nervousness, irritability, restlessness)
Tremor (like hands shaking)
Dizziness (light-headed, faintness)
Weight gain
Blood pressure increased
Erectile problems and/or ejaculation problems
Vomiting
Sleepiness (sedation)
Headache (all kinds of headache)
Cold symptoms
Acid indigestion, acid reflux, sore stomach, heartburn
Decreased appetite
Respiratory disorder (Flu and flu symptoms, cough, breathing difficulties)
Infections (urine infection, viral infections, etc,)

Here are the 15 side effects that you have chosen,
Please score them from the least tolerable for patients to the most tolerable (the most acceptable side effects). Please, give the score 1 to the least tolerable and the score 5 to the most acceptable,
Be careful, you can give one "1", two "2" ,three "3", four "4" and five "5".

Have you ever suffered from depression? (personal history of depression)*

- ○ Yes
- ○ No
- ○ I prefer not to answer

Have you ever taken antidepressant for depression?*

- ○ Yes
- ○ No
- ○ I prefer not to answer

Here is the list of antidepressants. Please choose the ones you have taken.*

- ☐ Agomelatine (Valdoxan®, Melitor®, Thymanax®)
- ☐ Amitriptyline (Elavil®, Laroxyl®, Tryptanol®, Endep®, Elatrol®, Tryptizol®, Trepiline®, Redomex®)
- ☐ Amoxapine (Defanyl®)
- ☐ Citalopram (Seropram®, Celapram®, Celexa®, Ciazil®, Cilift®, Cipram®, Cipramil®,

Ciprapine®, Citabax®, Citalec®, Citaxin®, Citrol®, Ecosol®, Mepha®, Recital®, Talam®, Zentius®, Zetalo®)

- ☐ Clomipramine (Anafranil®)
- ☐ Desipramine (Pertofran®)
- ☐ Deroxat (Paroxetine®)
- ☐ Dosulépine (Prothiaden ®)
- ☐ Doxépine (Quitaxon®)
- ☐ Duloxetine (Cymbalta®, Yentreve®, Xeristar®, AriClaim®)
- ☐ Escitalopram (Seroplex®)
- ☐ Fluoxetine (Prozac®)
- ☐ Fluvoxamine (Floxyfral®)
- ☐ Imipramine (Tofranil®)
- ☐ Iproniazide (Marsilid®)
- ☐ Maprotiline (Ludiomil®)
- ☐ Mianserine (Athymil®)
- ☐ Minalcipran (Ixel®)
- ☐ Mirtazapine (Norset®)
- ☐ Moclamine (Moclobemide®)
- ☐ Nortiptyline (Sensoval®, Aventyl®, Pamelor®, Norpress®, Allegron®, Noritren®, Nortrilen®)
- ☐ Paroxetine (Deroxat®, Paxil®, Seroxat®)
- ☐ Phénelzine (Nardelzine®, Nardil®)
- ☐ Sertraline (Zoloft®)
- ☐ Tianeptine (Stablon®, Coaxi®l, Tatinol®)
- ☐ Timipramine (Surmontil®)
- ☐ Tranylcypromine (Parnate®)
- ☐ Trazodone (Tritticon®, Desyrekn®, Thombarn®, Trialodine®, Trazolan®)
- ☐ Venlafaxine (Effexor®)
- ☐ Viloxazine (Vivalan®)
- ☐ Vortioxetine (Brintellix ®)
- ☐ I have never taken any of these drugs
- ☐ I have taken another antidepressants, Please specify : ______

While taking antidepressants, have you experienced any adverse effects?*

- ○ Yes Please specify : ______
- ○ No
- ○ I prefer not to answer

Could you please describe these side effects and their impacts on your life, in regards with the potential benefits of the treatment?

Supplementary material 5: Calibration of the weighted data set

The weighted data set was calibrated on gender, age and education according to the European Health Interview Survey

Characteristics	Patient Sample of SUSANA	Depressed sample of the European Health Interview Survey (n=15 575 over N=258 888) Weighted proportions	Weighted patient sample of SUSANA
Gender			
Male	21%	34%	34%
Female	79%	66%	66%
Age			
15-29	28%	16%	16%
30-44	36%	19%	19%
45-59	28%	29%	29%
>=60	8%	36%	37%
Education			
Primary or lower	4%	17%	17%
Secondary	59%	66%	66%
Tertiary	38%%	17 %	17%

Torre JA la, Vilagut G, Ronaldson A, Serrano-Blanco A, Martín V, Peters M, et al. Prevalence and variability of current depressive disorder in 27 European countries: a population-based study. Lancet Public Health. 1 oct 2021;6(10):e729-38.

Supplementary material 6 Further characteristics of patients

Characteristics of patients	Number (%) N=1631	Missing data
**Languages**		0
English	651 (39.9)	
French	948 (58.1)	
German	32 (2.0)	
**Countries (44)**		16 (1)
France	797 (48.9)	
UK	402 (24.7)	
USA	111 (6.8)	
Canada	107 (6.6)	
Germany	33 (2.0)	
Belgium	30 (1.8)	
Australia	21 (1.3)	
Switzerland	12 (0.7)	
Portugal	9 (0.6)	
Austria	8 (0.5)	
Irland	8 (0.5)	
Brazil	7 (0.4)	
Other (32 countries)*	85 (4.2)	
**Report being depressed**		
Yes	810 (49.7)	
No	621 (38.1)	
Don’t know	200 (12.3)	
**Type of antidepressants taken lifelong**		
Fluoxetine	638 (39.1)	
Venlafaxine	628 (38.5)	
Sertraline	602 (36.9)	
Citalopram	577 (35.4)	
Escitalopram	508 (31.2)	
Paroxetine	402 (24.7)	
Mirtazapine	262 (16.1)	
Amitryptiline	238 (14.6)	
Duloxetine	233 (14.3)	
clomipramine	112 (6.9)	
trazodone	106 (6.5)	
vortioxetine	86 (5.3)	
bupropion	76 (4.7)	
mianserine	66 (4.1)	
agomelatine	44 (2.7)	
tianeptine	39 (2.4)	
imipramine	38 (2.3)	
minalcipran	29 (1.8)	
fluvoxamine	21 (1.3)	
nortriptyline	20 (1.2)	
dosulepine	19 (1.2)	
doxepine	15 (0.9)	
maprotiline	15 (0.9)	
phénelzine	15 (0.9)	

timiptamine	14 (0.9)	
amoxapine	12 (0.7)	
moclamine	12 (0.7)	
lofepramine	7 (0.4)	
desipramine	5 (0.3)	
tranycypromine	5 (0.3)	
viloxazine	4 (0.2)	
iproniazide	3 (0.2)	
selegiline	3 (0.2)	
reboxetin	2 (0.1)	
nefazodone	2 (0.1)	
amineptine	2 (0.1)	
vilazodone	2 (0.1)	
isocarboxcine	1 (0.1)	
moclobemide	1 (0.1)	
opipramol	1 (0.1)	

*For sake of clarity we reported in the table the countries with >5 participants. Other countries by order : New Zealand, Sweden, South Africa, Morocco, Finland, India, Italy, Netherlands, Poland, Serbia, Tunisia, Spain, Mexico, Venezuela, Denmark, Greece, Israel, Malta, Norway, Pakistan, Peru, Rumania, Singapore, Turkey, Bhutan, Chili, Algeria, Croatia, Indonesia, Japan, Saudi Arabia, Lebanon.

Chevance A, *et al. Evid Based Ment Health* 2022; 25:e41–e48. doi: 10.1136/ebmental-2021-300418

Supplementary Material 7: Further characteristics of healthcare professionals

Characteristics of HCPs	Number (%) N=281
**Languages**	
English	115 (40.9)
French	153 (54.5)
German	13 (4.6)
**Countries (27)**	
France	130 (46.3)
UK	46 (16.4)
Netherland	22 (7.8)
Italie	17 (6.1)
Austria	12 (4.3)
Tunisia	6 (2.1)
Algeria	6 (2.1)
Switzerland	5 (1.8)
Brasil	5 (1.8)
USA	5 (1.8)
Other *	25(8.9)
NA	2 (0.7)
**Antidepressants taken life long (N=63)**	
paroxetine	21 (33.3)
citalopram	19 (30.2)
escitalopram	16 (25.4)
sertraline	14 (22.2)
fluoxetine	13 (20.6)
venlafaxine	9 (14.3)
**Agomelatine**	6 (9.5)
Amitryptiline	6 (9.5)
Clomipramine	5 (7.9)
Duloxetine	5 (7.9)
Mirtazapine	3 (4.7)
Amoxapine	2 (3.2)
Mianserine	2 (3.2)
Vortioxetine	2 (3.2)
Dosulepine	1 (1.6)
Doxepine	1 (1.6)
Fluvoxamine	1 (1.6)
Moclamine	1 (1.6)
timipramine	1 (1.6)
Trazodone	1 (1.6)
Non of them	2 (3.2)
other	5 (7.9)

*(Japan, Madagascar, Maroc, Australia, Portugal, Congo, Sweden, Danemark, Germany, Spain, Finland, Greece, Angola, India, Saudia Arabia)

Chevance A, *et al. Evid Based Ment Health* 2022; 25:e41–e48. doi: 10.1136/ebmental-2021-300418

Supplementary Material 8: Rankings of adverse events of antidepressants by patients using different analysis' methods

Table : Rankings of adverse events of antidepressants by patients using different analysis' methods: logit models, mean rank, top 1, top 3, and top 6

Name of the adverse events (AEs)	Logit model Probability of ranking the AE above the reference (cold symptoms)	Mean rank	Top1 (% of patients who ranked the AE as the most troublesome AE)	Top 3 (% of patients who ranked the AE in as one of the three most troublesome AEs)	Top 6 (% of patients who ranked the AE in as one of the six most troublesome AEs)
Insomnia	95.89 [95.16; 96.52]	3.58	11.5	32.8	50.3
Anxiety	95.17 [94.32; 95.91]	3.8	11	24.5	42
Fatigue	94.58 [93.63; 95.40]	4.01	5.2	19.4	39.1
Weight gain	93.16 [91.97; 94.18]	4.28	8.8	17.3	28.8
Agitation	92.72 [91.47; 93.80]	4.46	3.4	10.7	23.4
Sexual dysfunction	92.65 [91.37; 93.74]	4.26	8	18.8	35.1
Dizziness	92.53 [91.24; 93.65]	4.35	3.4	13.4	33.6
Headache	91.82 [90.41; 93.03]	4.42	4.9	15.9	30.2
Sleepiness	91.50 [90.04; 92.76]	4.44	4.4	14.5	31.3
Nausea	90.35 [88.70; 91.77]	4.55	3.7	15.3	29.1
Palpitations	89.60 [87.86; 91.12]	4.84	2.6	7.6	16.1
Sweating	89.54 [87.79; 91.07]	4.8	1.7	7.1	18.7
Vision disorder	89.18 [87.37; 90.75]	4.88	2	6.4	15.1
Vomiting	88.48 [86.56 ; 90.17]	4.71	6.3	13.3	22.3
Pain	88.40 [86.47; 90.08]	4.88	1.8	6.7	16.9
Tremor	88.28 [86.34; 89.98]	4.92	1.2	5.7	15.5
Stomach pain	87.92 [85.94;89.66]	4.99	1	4.5	12.4
Sore stomach	87.65 [85.61; 89.43]	5	1.5	5.3	13
Abnormal dreams	87.41 [85.34; 89.21]	5.07	2.2	5.8	10.9
Diarrhoea	87.21 [85.10; 89.06]	4.83	2.8	10.7	22.6
Hypotension	84.66[82.21; 86.82]	5.18	2.6	5.7	10.9

Infections	84.57 [82.08; 86.75]	5.11	2.9	8.3	13.5
Constipation	84.18 [81.65; 86.43]	5.13	0.7	5	14.9
Dry mouth	83.08 [80.39; 85.44	5.17	2.3	7.4	13.8
Respiratory disorder	82.70 [79.92; 85.12]	5.15	1.2	6.5	15.2
Hypertension	80.16 [77.06; 82.88]	5.37	0.8	3.4	8.4
Erectile disorder	73.40 [69.60; 76.96]	5.47	1.5	4.9	9.1
Decreased appetite	63.64 [58.85; 68.15]	5.7	0.4	1.5	3.8
Weight loss	49.75 [44.13; 55.36]	5.84	0.2	0.7	1.5
Cold symptoms	*		0.2	0.9	2.6

Mean rank: the ranking task consisted in ordering 30AEs in 6 ranks. Rank one corresponds to the most troublesome AE, rank 2 correspond to the two next troublesome AEs, etc, and rank 6 the less troublesome AEs.

Top X: correspond to the X AEs participants considered the most troublesome. For instance, 11.5% of the patients ranked insomnia as the most troublesome AE.

AEs are presented in the order of the ranking provided by the logit model (the probability of ranking a given AE above the reference, cold symptoms)

Supplementary Material 9: Comparison of the ranking men and women

Adverse events	Probability [95%CI] of patients	Probability [95%CI] of women	Probability [95%CI] of men	p-value	Rank difference (women-men)
**Insomnia**	95.89 [95.16; 96.52]	96.23 [95.43; 96.88]	94.12 [91.87; 95.78]	0.02	-1
**Anxiety**	95.17 [94.32; 95.91]	95.39 [94.44; 96.20]	94.22 [92.00; 95.83]	0.24	1
**Fatigue**	94.58 [93.63; 95.40]	95.07 [94.05; 95.93]	92.00 [89.01; 94.22]	0.01	-1
**Weight gain**	93.16 [91.97; 94.18]	94.22 [93.06; 95.22]	86.84 [82.14; 90.29]	<0.0001	-6
**Agitation**	92.72 [91.47; 93.80]	93.24 [91.87; 94.38]	89.90 [86.30; 92.70]	0.03	-1
**Sexual dysfunction**	92.65 [91.37; 93.74]	92.65 [91.15; 93.87]	93.24 [90.65; 95.12]	0.65	5
**Dizziness**	92.53 [91.24; 93.65]	93.20 [91.80; 94.35]	88.89 [84.85; 91.94]	0.001	-1
**Headache**	91.82 [90.41; 93.03]	92.65 [91.15; 93.90]	87.34 [83.05; 90.83]	0.004	-1
**Sleepiness**	91.50 [90.04; 92.76]	92.25 [90.65; 93.59]	87.34 [82.76; 90.74]	0.01	0
**Nausea**	90.35 [88.70; 91.77]	91.38 [89.69; 92.86]	84.13 [78.72; 88.24]	0.001	-6
**Palpitations**	89.60 [87.86; 91.12]	90.48 [88.64; 92.13]	84.85 [79.59; 88.89]	0.01	-2
**Sweating**	89.54 [87.79; 91.07]	90.65 [88.76; 92.25]	83.33 [77.78; 87.80]	0.002	-6
**Vision disorder**	89.18 [87.37; 90.75]	89.80 [87.80; 91.53]	85.71 [80.77; 89.47]	0.06	2
**Vomiting**	88.48 [86.56; 90.17]	89.47 [97.34; 91.23]	81.82 [76.19; 86.67]	0.003	-6
**Pain**	88.40 [86.47; 90.08]	89.25 [87.18; 90.99]	84.13 [78.72; 88.24]	0.03	0
**Tremor**	88.28 [86.34; 89.98]	89.01 [86.84; 90.83]	85.07 [80.00; 89.01]	0.09	3
**Stomach pain**	87.92 [85.94; 89.66]	89.01 [86.84; 90.83]	82.46 [76.74; 87.01]	0.01	-2
**Sore stomach**	87.65 [85.61; 89.43]	88.51 [86.30; 90.38]	82.46 [76.74; 87.01]	0.02	1
**Abnormal dreams**	87.41 [85.34; 89.21]	88.51 [86.30; 90.38]	81.48 [75.61; 86.30]	0.01	-3
**Diarrhoea**	87.21 [85.10; 89.06]	87.50 [85.07; 89.58]	85.51 [80.39; 89.36]	0.42	8
**Hypotension**	84.66[82.21; 86.82]	85.71 [83.05; 88.10]	77.78 [70.59; 83.33]	0.01	-3
**Infections**	84.57 [82.08; 86.75]	85.71 [83.05; 87.95]	76.74 [69.70; 82.76]	0.01	-3
**Constipation**	84.18 [81.65; 86.43]	85.29 [82.46; 87.65]	79.17 [72.22; 84.62]	0.05	1
**Dry mouth**	83.08 [80.39; 85.44	84.38 [81.48; 87.01]	76.74 [69.70; 82.76]	0.02	-2
**Respiratory disorder**	82.70 [79.92; 85.12]	83.33 [80.00; 85.92]	77.78 [70.59; 83.61]	0.1	2
**Hypertension**	80.16 [77.06; 82.88]	80.77 [77.27; 83.87]	76.19 [68.75; 82.46]	0.22	-1
**Erectile disorder**	73.40 [69.60; 76.96]	56.52. [50.0; 62.96]	91.38 [88.24; 93.75]	<0.0001	23
**Decreased appetite**	63.64 [58.85; 68.15]	65.52 [60.00; 69.70]	56.52 [47.37; 66.67]	0.18	-1
**Weight loss**	49.75 [44.13; 55.36]	50.00 [44.44;56.52]	47.37 [37.50; 58.33]	0.78	0
**Cold symptoms**	*	*	*	*	*

Table. Comparison of the ranking of women and men by ordering the estimates of the logit model for ranked item using the raw data set. *We report the p-value testing the interaction of each AE with gender. Rank difference: we substracted for each AE the rank number of men to the rank number of women. For instance, insomnia was ranked in the first place by women and in the second place by men which gives a difference of -1 rank. Bonferroni threshold = 0.0017*

Mean rank (absolute value) difference: 3.2

Supplementary Material 10: Comparison of the ranking of patients with mild and severe depression

Adverse events	Probability [95%CI] of patients	Probability [95%CI] of patients with mild depression	Probability [95%CI] of patients with severe depression	p-value	Rank difference (mild-severe)
**Insomnia**	95.89 [95.16; 96.52]	95.85 [94.87; 96.63]	95.97 [94.74 ;96.92]	0.86	0
**Anxiety**	95.17 [94.32; 95.91]	95.24 [94.15; 96.14]	95.07 [93.59 ;96.24]	0.84	-1
**Fatigue**	94.58 [93.63; 95.40]	94.05 [92.70; 95.15]	95.41[94.01 ;96.49]	0.13	1
**Weight gain**	93.16 [91.97; 94.18]	93.10 [91.60 ; 94.38]	93.24[91.23 ;94.79]	0.93	1
**Agitation**	92.72 [91.47; 93.80]	92.59 [90.99 ; 94.01]	92.91 [90.83 ;94.57]	0.81	2
**Sexual dysfunction**	92.65 [91.37; 93.74]	93.15 [91.60 ;94.41]	91.74 [89.36 ;93.67]	0.28	-4
**Dizziness**	92.53 [91.24; 93.65]	92.65 [90.99 ;94.01]	92.37 [90.10 ;94.15]	0.84	0
**Headache**	91.82 [90.41; 93.03]	92.00 [90.29 ;93.51]	91.45 [88.89 ;93.42]	0.68	-1
**Sleepiness**	91.50 [90.04; 92.76]	91.38 [89.47 ;92.96]	91.74 [89.36 ;93.67]	0.78	2
**Nausea**	90.35 [88.70; 91.77]	90.57 [88.51 ;88.51]	89.90 [87.01 ;92.25]	0.70	-1
**Palpitations**	89.60 [87.86; 91.12]	89.47 [87.18 ; 91.38]	89.80 [86.84 ;92.06]	0.89	0
**Sweating**	89.54 [87.79; 91.07]	89.25 [86.84 ;91.15]	90.10 [87.18 ;92.31]	0.61	4
**Vision disorder**	89.18 [87.37; 90.75]	89.25 [87.01 ;91.23]	89.01 [85.92 ;91.53]	0.89	0
**Vomiting**	88.48 [86.56; 90.17]	90.10 [87.95 ;91.94]	85.07 [80.77 ;88.37]	0.01	-11
**Pain**	88.40 [86.47; 90.08]	88.51 [86.11 ;90.57]	88.24 [84.85 ;90.91]	0.89	0
**Tremor**	88.28 [86.34; 89.98]	88.10 [85.51 ;85.29]	88.51 [85.29 ;91.15]	0.83	2
**Stomach pain**	87.92 [85.94; 89.66]	87.95 [85.29 ;90.10]	87.95 [84.62 ;90.65]	0.99	2
**Sore stomach**	87.65 [85.61; 89.43]	88.10 [85.51 ;90.20]	86.84 [83.05 ;89.80]	0.52	-1
**Abnormal dreams**	87.41 [85.34; 89.21]	87.18 [84.38 ;89.47]	87.65 [84.38 ;90.48]	0.79	3
**Diarrhoea**	87.21 [85.10; 89.06]	87.80 [85.29 ;90.10]	86.11 [82.14 ;89.25]	0.39	0
**Hypotension**	84.66[82.21; 86.82]	84.38 [81.13 ;87.18]	85.07 [81.13 ;88.37]	0.78	1
**Infections**	84.57 [82.08; 86.75]	85.92 [83.05 ;88.37]	81.82 [77.27 ;85.92]	0.11	-3
**Constipation**	84.18 [81.65; 86.43]	84.13 [80.77 ;87.01]	84.13 [80.00 ;87.65]	0.97	0
**Dry mouth**	83.08 [80.39; 85.44	81.13 [77.27 ;84.38]	85.71 [81.82 ;88.89]	0.08	5
**Respiratory disorder**	82.70 [79.92; 85.12]	83.33 [80.00 ;86.30]	81.82 [76.74 ;85.71]	0.56	-1
**Hypertension**	80.16 [77.06; 82.88]	80.77 [77.27 ; 84.13]	78.72 [72.97 ;83.33]	0.49	0
**Erectile disorder**	73.40 [69.60; 76.96]	75.61 [71.43 ;79 ;59]	68.75 [61.54 ;75.00]	0.09	-1
**Decreased appetite**	63.64 [58.85; 68.15]	58.33 [52.38 ;65.52]	69.70 [62.96 ;75.61]	0.03	1
**Weight loss**	49.75 [44.13; 55.36]	47.37 [41.18 ;54.55]	54.55 [44.44 ;61.54]	0.28	1
**Cold symptoms**	*	*	*	*	*

Table. Comparison of the ranking of patients with mild (PHQ-9 score <15) and severe (PHQ-9 score ≥ 15) depression at the time of the survey, by ordering the estimates of the logit model for ranked item, using the raw data set. *We report the p-value testing the interaction of each AE with the severity of the disease. Rank difference: we substracted for each AE the rank number of patients with severe depression to the rank number of patients with mild depression. For instance, anxiety was ranked in the second place by patients with mild depression and in the third place by patients with severe depression which gives a difference of -1 rank. Bonferroni threshold = 0.0017*

Mean rank (absolute value) difference: 1.6

Supplementary Material 11: Comparison of the ranking of patients with current and previous antidepressant medication.

Adverse events	Probability [95%CI] of patients	Probability [95%CI] of patients with current medication	Probability [95%CI] of patients with previous medication	p-value	Rank difference
**Insomnia**	95.89 [95.16; 96.52]	96.31 [94.92 ;97.33]	95.73 [94.82 ;96.48]	0.44	0
**Anxiety**	95.17 [94.32; 95.91]	95.69 [94.08 ;96.87]	94.97 [93.94 ;95.85]	0.42	0
**Fatigue**	94.58 [93.63; 95.40]	94.59 [92.59 ;96.06]	94.62 [93.51 ;95.56]	0.97	0
**Weight gain**	93.16 [91.97; 94.18]	93.75 [91.53 ;95.45]	92.91 [91.45 ;94.15]	0.50	-1
**Agitation**	92.72 [91.47; 93.80]	93.51 [91.15 ;95.26]	92.42 [90.83 ;93.71]	0.40	-2
**Sexual dysfunction**	92.65 [91.37; 93.74]	94.12 [91.94 ;95.71]	92.00 [90.38 ;93.33]	0.10	3
**Dizziness**	92.53 [91.24; 93.65]	93.71 [91.45 ;95.43]	92.00 [90.38 ;93.38]	0.20	0
**Headache**	91.82 [90.41; 93.03]	92.65 [90.00 ;94.62]	91.53 [89.80 ;92.96]	0.44	-1
**Sleepiness**	91.50 [90.04; 92.76]	92.75 [90.10 ;94.71]	90.99 [89.13 ;92.48]	0.23	1
**Nausea**	90.35 [88.70; 91.77]	91.94 [89.01 ;94.12]	89.58 [87.50 ;91.38]	0.17	0
**Palpitations**	89.60 [87.86; 91.12]	91.87 [89.01 ;94.05]	88.51 [86.30 ;90.48]	0.06	1
**Sweating**	89.54 [87.79; 91.07]	89.47 [85.92 ;92.31]	89.58 [87.50 ;91.38]	0.96	-6
**Vision disorder**	89.18 [87.37; 90.75]	91.74 [88.76 ;93.94]	87.95 [85.71 ;90.00]	0.04	1
**Vomiting**	88.48 [86.56; 90.17]	90.83 [87.65 ;93.29]	87.34 [84.85 ;89.47]	0.07	3
**Pain**	88.40 [86.47; 90.08]	89.47 [85.92 ;92.31]	87.95 [85.51 ;90.00]	0.44	-2
**Tremor**	88.28 [86.34; 89.98]	90.57 [87.18 ;93.10]	87.18 [84.85 ;89.36]	0.09	3
**Stomach pain**	87.92 [85.94; 89.66]	89.69 [86.11 ;92.48]	87.18 [84.62 ;89.25]	0.21	4
**Sore stomach**	87.65 [85.61; 89.43]	88.24 [84.13 ;91.30]	87.50 [85.07 ;89.58]	0.72	-4
**Abnormal dreams**	87.41 [85.34; 89.21]	89.01 [85.07 ;91.87]	86.67 [84.13 ;88.89]	0.31	2
**Diarrhoea**	87.21 [85.10; 89.06]	87.34 [83.05 ;90.74]	87.18 [84.62 ;89.36]	0.92	-3
**Hypotension**	84.66[82.21; 86.82]	87.80 [83.61 ;78.26]	83.33 [80.00 ;85.92]	0.07	3
**Infections**	84.57 [82.08; 86.75]	87.01 [82.46 ;90.38]	83.61 [80.39 ;86.11]	0.18	0
**Constipation**	84.18 [81.65; 86.43]	85.29 [80.39 ;89.13]	83.87 [80.77 ;86.49]	0.56	-3
**Dry mouth**	83.08 [80.39; 85.44	83.61 [78.26 ;87.95]	82.76 [79.59 ;85.71]	0.78	-1
**Respiratory disorder**	82.70 [79.92; 85.12]	85.71 [81.13 ;89.47]	81.13 [77.78 ;84.38]	0.11	2
**Hypertension**	80.16 [77.06; 82.88]	81.82 [76.19 ;86.49]	79.59 [75.61 ;82.76]	0.47	0
**Erectile disorder**	73.40 [69.60; 76.96]	78.72 [72.22 ;84.13]	70.59 [65.52 ;75.61]	0.05	0
**Decreased appetite**	63.64 [58.85; 68.15]	69.70 [61.54 ;77.27]	61.54 [54.55 ;66.67]	0.08	0
**Weight loss**	49.75 [44.13; 55.36]	60.00 [50.00 ;68.75]	44.44 [37.50 ;52.38]	0.02	1
**Cold symptoms**	*	*	*	*	*

Table. Comparison of the ranking of patients with current antidepressant medication at the time of the survey and patient with previous antidepressant medication, by ordering the estimates of the logit model for ranked item. *We report the p-value testing the interaction of each AE with the severity of the disease. Rank difference: we substracted for each AE the rank number of patients with previous medication to the rank number of patients with current medication. For instance, weight gain was ranked in the fourth place by patients with previous medication and in the fifth place by patients with current medication which gives a difference of -1 rank. Bonferroni threshold = 0.0017*

Mean rank (absolute value) difference: 1.6

Supplementary material 12 : Results of the sensitivity analysis

Adverse events	Probability 95% raw data set of patients with depression	Probability 95% weighted data set	Rank difference
**Insomnia**	95,82 [95,08;96,45]	95,86 [95,48;96,21]	0
**Anxiety**	95,10 [94,25;95,84]	95,49 [95,06;95,87]	0
**Fatigue**	94,49 [93,53;95,31]	95,03 [94,56;95,46]	0
**Weight gain**	93,08 [91,90;94,09]	91,33 [90,34;92,19]	-3
**Agitation**	92,60 [91,35;93,69]	92,4 [91,57;93,13]	0
**Sexual dysfunction**	92,49 [91,21;93,60]	92,48 [91,66;93,19]	2
**Dizziness**	92,44 [91,15;93,55]	91,35 [90,36;92,21]	1
**Headache**	91,65 [90,25;92,87]	88,04 [86,47;89,36]	-1
**Sleepiness**	91,38 [89,93;92,64]	91,03 [89,99;91,93]	1
**Nausea**	90,16 [88,52;91,59]	84,47 [82,14;86,38]	-11
**Palpitations**	89,51 [87,79;91,02]	87,43 [85,74;88,85]	-2
**Sweating**	89,40 [87,65;90,92]	87,03 [85,26;88,51]	-2
**Vision disorder**	88,98 [87,17;90,55]	86,69 [84,85;88,23]	-3
**Vomiting**	88,23 [86,30;89,92]	80,53 [77,18;83,18]	-12
**Pain**	88,17 [86,24;89,86]	87,63 [85,98;89,02]	4
**Tremor**	88,14 [86,21;89,84]	87,51 [85,83;88,91]	4
**Stomach pain**	87,71 [85,72;89,46]	85,54 [83,45;87,26]	-3
**Sore stomach**	87,46 [85,43;89,24]	85,87 [83,86;87,54]	-1
**Abnormal dreams**	87,27 [85,22;89,07]	87,7 [86,07;89,08]	9
**Diarrhoea**	87,02 [84,91;88,87]	86,32 [84,39;87,91]	2
**Hypotension**	84,48 [82,03;86,64]	80,8 [77,53;83,39]	-3
**Infections**	84,35 [81,88;86,54]	77,39 [73,12;80,69]	-5
**Constipation**	83,91 [81,38;86,16]	83,69 [81,17;85,73]	1
**Dry mouth**	82,92 [80,26;85,29]	86,7 [84,86;88,23]	9
**Respiratory disorder**	82,37 [79,63;84,81]	82,3 [79,43;84,6]	2
**Hypertension**	79,88 [76,84;82,62;]	80,8 [77,52;83,39]	1
**Erectile disorder**	73,07[69,22;76,60]	86,57 [84,7;88,12]	10
**Decreased appetite**	64,08 [59,39;68,51]	69,36 [62,04;74,61]	0
**Weight loss**	50,21 [44,69;55,71]	60,47 [48,7;68,26]	0
**Cold symptoms**	*	*	*

Table. Comparison of the ranking of the raw data set (1660 patients with reported depression) and the weighted data set calibrated on age, sex and education according to the European Health Survey. The estimates of the logit model for ranked item. *Rank difference: we substracted for each AE the rank number of the raw data set to the rank numbers of the weighted data set .*

Mean rank difference: 3.2

Supplementary material 13: Rankings of adverse events of antidepressants by HCPs using different analysis' methods

Table : Rankings of adverse events of antidepressants by HCPs using different analysis' methods: logit models, mean rank, top 1, top 3, top 6 and top15

Name of the adverse events (AEs)	Probability of ranking the AE above the reference (cold symptoms)	Mean rank	Top1 (% of HCPs who ranked the AE as the most troublesome AE)	Top 3 (% of HCPs who ranked the AE in as one of the three most troublesome AEs)	Top 6 (% of HCPs who ranked the AE in as one of the six most troublesome AEs)	Top 15 (% of HCPs who ranked the AE in as one of the 15 most troublesome AEs)
Insomnia	98.29 [96.56; 99.15]	4.04	5.0	19.9	42.9	69.5
Anxiety	97.48 [94.98; 98.75]	4.47	5.7	13.1	25.5	61.3
Fatigue	97.85 [95.71; 98.94]	4.48	2.1	6.4	22.7	69.8
Weight gain	98.85 [97.68; 99.43]	3.65	12.1	28.1	45.1	86.6
Agitation	98.44 [96.86; 99.23]	3.96	12.8	23.4	34.7	78.7
Sexual dysfunction	99.23 [98.45; 99.62]	3.16	18.1	38.7	58.2	91.1
Dizziness	98.06 [96.13; 99.04]	4.32	2.5	11.7	30.8	73.0
Headache	97.34 [94.72; 98.68]	4.57	2.5	11.4	25.6	61.0
Sleepiness	98.79 [97.57; 99.40]	3.74	6.4	23.4	45.0	83.3
Nausea	98.31 [96.61; 99.16]	4.1	4.3	18.5	37.6	74.5
Palpitations	94.90 [90.05; 97.46]	5.22	2.1	6.0	9.2	41.8
Sweating	97.20 [94.44; 98.61]	4.7	1.8	7.8	18.1	61.3
Vision disorder	95.67 [91.50; 97.84]	5.09	2.5	5.3	12.0	46.7
Vomiting	94.07 [88.47; 97.04]	5.25	0.7	5.3	9.9	35.4
Pain	85.86 [74.01; 92.82]	5.7	0.0	1.1	3.2	15.9
Tremor	96.96 [93.97; 98.49]	4.84	1.1	4.6	15.6	59.6
Stomach pain	95.66 [91.49; 97.84]	5.14	1.8	4.3	9.6	47.9
Sore stomach	91.88 [84.47; 95.92]	5.54	0.4	1.5	4.0	28.5
Abnormal dreams	96.83 [93.74; 98.42]	4.95	2.1	4.9	12.0	61.6
Diarrhoea	94.95 [90.12; 97.48]	5.13	1.1	4.6	13.5	40.1

Hypotension	96.35 [92.79; 98.18]	4.89	2.5	10.7	16.4	52.2
Infections	66.99 [46.38; 82.64]	5.88	0.4	0.8	2.2	5.7
Constipation	95.69 [91.52; 97.85]	4.99	1.1	6.1	19.2	44.7
Dry mouth	97.54 [95.11; 98.78]	4.54	2.5	9.9	26.9	66.3
Respiratory disorder	68.42 [48.22; 83.45]	5.88	0.0	0.7	2.5	6.1
Hypertension	88.75 [78.91; 94.33]	5.53	2.5	5.0	8.5	19.9
Erectile disorder	98.84 [97.66; 99.43]	3.73	6.0	26.2	43.6	88.3
Decreased appetite	88.16 [77.92; 94.01]	5.69	0.4	0.4	2.5	19.5
Weight loss	70.75 [51.32; 84.73]	5.87	0.0	0.4	1.8	6.7
Cold symptoms	*	5.93	0.0	0.7	1.8	2.9

Mean rank: the ranking task consisted in ordering 30AEs in 6 ranks. Rank one corresponds to the most troublesome AE, rank 2 correspond to the two next troublesome AEs, etc, and rank 6 the less troublesome AEs.

Top X: correspond to the X AEs HCPs considered the most troublesome. For instance, 5% of the HCPs ranked insomnia as the most troublesome AE.

*AEs are presented in the order of the ranking of the **patient**s provided by the logit model (the probability of ranking a given AE above the reference, cold symptoms)*

Supplementary material 14: Comparison of the ranking of patients and HCPs

Adverse events	Probability [95%CI] of patients	Probability [95%CI] of HCPs	p-value	Rank difference (patients-HCPs)
**Insomnia**	95.89 [95.16; 96.52]	98.29 [96.56; 99.15]	0.02	-6
**Anxiety**	95.17 [94.32; 95.91]	97.48 [94.98; 98.75]	0.07	-9
**Fatigue**	94.58 [93.63; 95.40]	97.85 [95.71; 98.94]	0.01	-6
**Weight gain**	93.16 [91.97; 94.18]	98.85 [97.68; 99.43]	<0.0001	2
**Agitation**	92.72 [91.47; 93.80]	98.44 [96.86; 99.23]	<0.0001	0
**Sexual dysfunction**	92.65 [91.37; 93.74]	99.23 [98.45; 99.62]	<0.0001	5
**Dizziness**	92.53 [91.24; 93.65]	98.06 [96.13; 99.04]	0.0002	-1
**Headache**	91.82 [90.41; 93.03]	97.34 [94.72; 98.68]	0.002	-4
**Sleepiness**	91.50 [90.04; 92.76]	98.79 [97.57; 99.40]	<0.0001	5
**Nausea**	90.35 [88.70; 91.77]	98.31 [96.61; 99.16]	<0.0001	4
**Palpitations**	89.60 [87.86; 91.12]	94.90 [90.05; 97.46]	0.04	-10
**Sweating**	89.54 [87.79; 91.07]	97.20 [94.44; 98.61]	0.0002	-1
**Vision disorder**	89.18 [87.37; 90.75]	95.67 [91.50; 97.84]	0.01	-5
**Vomiting**	88.48 [86.56 ; 90.17]	94.07 [88.47; 97.04]	0.06	-8
**Pain**	88.40 [86.47; 90.08]	85.86 [74.01; 92.82]	0.58	-11
**Tremor**	88.28 [86.34; 89.98]	96.96 [93.97; 98.49]	0.0001	2
**Stomach pain**	87.92 [85.94;89.66]	95.66 [91.49; 97.84]	0.003	-2
**Sore stomach**	87.65 [85.61; 89.43]	91.88 [84.47; 95.92]	0.23	-5
**Abnormal dreams**	87.41 [85.34; 89.21]	96.83 [93.74; 98.42]	<0.0001	4
**Diarrhoea**	87.21 [85.10; 89.06]	94.95 [90.12; 97.48]	0.01	0
**Hypotension**	84.66[82.21; 86.82]	96.35 [92.79; 98.18]	<0.0001	5
**Infections**	84.57 [82.08; 86.75]	66.99 [46.38; 82.64]	0.03	-7
**Constipation**	84.18 [81.65; 86.43]	95.69 [91.52; 97.85]	0.0002	6
**Dry mouth**	83.08 [80.39; 85.44	97.54 [95.11; 98.78]	<0.0001	14
**Respiratory disorder**	82.70 [79.92; 85.12]	68.42 [48.22; 83.45]	0.07	-3
**Hypertension**	80.16 [77.06; 82.88]	88.75 [78.91; 94.33]	0.09	2
**Erectile disorder**	73.40 [69.60; 76.96]	98.84 [97.66; 99.43]	<0.0001	24
**Decreased appetite**	63.64 [58.85; 68.15]	88.16 [77.92; 94.01]	0.0002	3
**Weight loss**	49.75 [44.13; 55.36]	70.75 [51.32; 84.73]	0.02	3
**Cold symptoms**	*	*	*	*

Table. Comparison of the ranking of patients and HCPs by ordering the estimates of the logit model for ranked item using the raw data set. *We report the p-value testing the interaction of each AE with status of participants. Rank difference: we substracted for each AE the rank number of HCPs to the rank number of patients. For instance. insomnia was ranked in the first place by patients and in the seventh place by HCPs which gives a difference of -6 rank. Bonferroni threshold= 0,0017*

Mean rank (absolute value) difference: 5.4

Chevance A, *et al. Evid Based Ment Health* 2022; 25:e41–e48. doi: 10.1136/ebmental-2021-300418

Supplementary Material 15: Adverse events identified with the qualitative content analysis

Over a total of 102 adverse events reported by patients: 66 were not included in the ranking task table A) and 36 were (table B).

**Adverse events not proposed in the ranking task**	**Citations (%)<br>N=1323 (1283 patients. 40HCPs)**
**Auto-aggressivity**	
New suicidal ideation	62 (4.7)
Increase of suicidal ideation	28 (2.1)
Suicidal attempts	18 (1.4)
Self-harm	11 (0.8)
**Psychiatric effects**	
Emotional numbing/detachment	114 (8.6)
Irritability	79 (6)
Dissociative symptoms	37 (2.8)
Mood swings	32 (2.4)
Mania - hypomania	21 (1.6)
To feel weird	9 (0.7)
Paranoiac ideation	7 (0.5)
Antidepressants changed me (self-recognition)	5 (0.4)
Impulsivity	17 (1.3)
Obsessional ideas	5 (0.4)
Eating disorder	2 (0.2)
**Sleep disturbances**	
Other sleep disturbances	70 (5.3)
Snorring/sleep apnea	4 (0.3)
**Cognitive effects**	
Trouble with concentration	101 (7.6)
Trouble with memory	47 (3.6)
Cognitive impairement	19 (1.4)
Paranoid ideation	7 (0.5)
Cognitive bias	7 (0.5)
Flashbacks	2 (0.2)
**Neurological and neurovegetative effects**	
Hot flushes	17 (1.3)
Paresthesia	16 (1.2)
Yawning	8 (0.6)
Fall	6 (0.5)
Seizures	6 (0.5)
Balance problems	2 (0.2)
Tic	2 (0.2)
Thrill	1 (0.1)
**Motor and muscular effects**	
Restless leg	22 (1.7)
Cramp	21 (1.6)
Slowness	15 (1.1)
Leg twitch	12 (0.9)
Bruxism	12 (0.9)

Mucle weakness	8 (0.6)
Stuttering	8 (0.6)
**Pain**	
« Brain zap »	29 (2.2)
**Metabolic and endocrine effects**	
Increased appetite	59 (4.5)
Intolerance to alcohol	4 (0.3)
Menstrual disorder	4 (0.3)
Hyperprolactinemia	2 (0.2)
Change in insulin sensitivity/diabetes	2 (0.2)
Liver disorder	2 (0.2)
**Digestive effects**	
Dental problem	4 (0.3)
Bad taste in mouth	4 (0.3)
**Cardiovascular effects**	
Oedema	7 (0.5)
Heavy legs	2 (0.2)
Paleness	2 (0.2)
Extreme cold extremities	1 (0.1)
**Ophtalmological and ENT effects**	
Tinnitus	10 (0.8)
Dry eyes	6 (0.5)
Hyperacusis	4 (0.3)
Glaucoma	2 (0.2)
**Dermatological effects**	
Itching	12 (0.9)
Hypersensitivity to antidepressant	8 (0.6)
Vagina dryness	6 (0.5)
Dry eyes	6 (0.5)
Sun sensitivity	5 (0.4)
Hair loss	4 (0.3)
Psoriasis	1 (0.1)
**Other**	
Withdrawal symptoms	78 (5.9)
Urinary problems	21 (1.6)
Bruising	7 (0.5)
Serotonin syndrom	3 (0.2)

Table A. Adverse events identified with the qualitative content analysis of the 1323 responses to the open-ended questions not proposed in the ranking.

Chevance A, et al. Evid Based Ment Health 2022; 25:e41–e48. doi: 10.1136/ebmental-2021-300418

Adverse events proposed in the ranking task	Citations (%) N=1323 (1283 patients, 40HCPs)
Anxiety	202 (15.3)
Insomnia	145 (11)
Abnormal dreams	74 (5.6)
Nightmares	62 (4.7)
Sedation	235 (17.8)
Dizziness	170 (12.8)
Sweating	160 (12.1)
Tremor	125 (9.4)
Fatigue/lack of energy	286 (21.6)
Agitation	80 (6.0)
Reduced libido	160 (12.1)
Orgasm disorder	102 (7.7)
Sexual dysfunction (not specified)	90 (6.8)
Erectile dysfunction	42 (3.2)
No sex drive	22 (1.7)
Headache	132 (10)
Abdominal pain	41 (3.1)
Other Pain (beside headache and abdominal pain)	34 (2.6)
Weight gain	363 (27.4)
Weight loss	52 (3.9)
Appetite loss	27 (2)
Nausea	235 (17.8)
Constipation	81 (6.1)
Diarrhea	73 (5.5)
Reflux gastritis	56 (4.2à
Vomiting	45 (3.4)
Irritable Bowel Syndrom	32 (2.4)
Palpitation	69 (5.2)
Hypotension	57 (4.3)
Faint	17 (1.3)
Hypertension	9 (0.7)
Vision disorder	46 (3.5)
Dry mouth	142 (10.7)
Cold/flu-like symptoms	11 (0.8)
Infections	3 (0.2)
Shortness of breath (respiratory disorder)	3 (0.2)

Table B. Adverse events identified with the qualitative content analysis of the 1323 responses to the open-ended question corresponding with those proposed in the ranking.

Chevance A, *et al. Evid Based Ment Health* 2022; 25:e41–e48. doi: 10.1136/ebmental-2021-300418

Themes	Number of participants	Proportion (1283 patients, 40 HCPs)
**Acceptability**		
Harm to benefit balance in favour of continuing medication	194	14.7
Short term duration of AEs	124	9.4
Clinical benefit of antidepressant	66	5
AEs considered as minor/tolerable	14	1.1
Balance harm/benefit was neutral	7	0.5
**Intolerability**		
Harm to benefit in favour of stopping medication	106	8
Symptoms of depression persisted or worsened on medication	89	6.7
AEs persist after medication stop	23	1.7
Efficacy rapidly stopped	12	0.9
**Type of consequences**		
Impact on daily living	151	11.4
Impairment in relationship with family and friends and more broadly with social life	100	7.6
Impairment in professional life	91	7.1
Lowering of self esteem	82	6.2
AEs led to emergency department	4	0.3
AEs led to hospitalization under section	2	0.2
**Coping strategies toward AEs**		
Treatment change resolved difficulties with AEs	80	6.1
Tapering off antidepressant to prevent AEs	16	1.2
AE with generic	6	0.5
AE led to weight management program/surgery	2	0.2
Adapting treatment regime due to AE	2	0.2
AEs require further medication to be managed	1	0.1
Use and misuse of illicit drugs/alcohol to alleviate AE	2	0.1
**AEs and the patient-HCP relationship**		
HCPs not interested in AE reported	8	0.6
Patients ask for more information on Aes	4	0.3
HCPs does not believe the patient regarding his/her reporting of AE	4	0.3
Changed psychiatrist because of AE	1	0.07
**Difficulty to distinguish symptoms due to antidepressant from other possible causes**		
Difficulty to distinguish between depressive symptoms and AEs	26	2
Difficulties to make a difference with other drugs	21	1.6
Difficulty to distinguish AEs from other health problem	26	0.9

Table C. Further themes identified by the qualitative content analysis of the open-ended question

Chevance A, *et al. Evid Based Ment Health* 2022; 25:e41–e48. doi: 10.1136/ebmental-2021-300418
